# Endo- and Exometabolome Crosstalk in Mesenchymal Stem Cells Undergoing Osteogenic Differentiation

**DOI:** 10.3390/cells11081257

**Published:** 2022-04-07

**Authors:** Daniela S. C. Bispo, Lenka Michálková, Marlene Correia, Catarina S. H. Jesus, Iola F. Duarte, Brian J. Goodfellow, Mariana B. Oliveira, João F. Mano, Ana M. Gil

**Affiliations:** 1 Department of Chemistry, University of Aveiro, CICECO—Aveiro Institute of Materials (CICECO/UA), 3810-193 Aveiro, Portugal; d.bispo@ua.pt (D.S.C.B.); marlene24@ua.pt (M.C.); catarina.jesus@ua.pt (C.S.H.J.); ioladuarte@ua.pt (I.F.D.); brian.goodfellow@ua.pt (B.J.G.); mboliveira@ua.pt (M.B.O.); jmano@ua.pt (J.F.M.); 2 Department of Analytical Chemistry, University of Chemistry and Technology, 166 28 Prague, Czech Republic; michalkova@icpf.cas.cz

**Keywords:** mesenchymal stem cells, osteogenic differentiation, osteogenesis, NMR spectroscopy, metabolomics, lipidomics, endometabolome, exometabolome

## Abstract

This paper describes, for the first time to our knowledge, a lipidome and exometabolome characterization of osteogenic differentiation for human adipose tissue stem cells (hAMSCs) using nuclear magnetic resonance (NMR) spectroscopy. The holistic nature of NMR enabled the time-course evolution of cholesterol, mono- and polyunsaturated fatty acids (including ω-6 and ω-3 fatty acids), several phospholipids (phosphatidylcholine, phosphatidylethanolamine, sphingomyelins, and plasmalogens), and mono- and triglycerides to be followed. Lipid changes occurred almost exclusively between days 1 and 7, followed by a tendency for lipidome stabilization after day 7. On average, phospholipids and longer and more unsaturated fatty acids increased up to day 7, probably related to plasma membrane fluidity. Articulation of lipidome changes with previously reported polar endometabolome profiling and with exometabolome changes reported here in the same cells, enabled important correlations to be established during hAMSC osteogenic differentiation. Our results supported hypotheses related to the dynamics of membrane remodelling, anti-oxidative mechanisms, protein synthesis, and energy metabolism. Importantly, the observation of specific up-taken or excreted metabolites paves the way for the identification of potential osteoinductive metabolites useful for optimized osteogenic protocols.

## 1. Introduction

Mesenchymal stem cells (MSCs) are undifferentiated non-hematopoietic cells that possess the ability of self-renewal and the potential to differentiate into multiple lineages (e.g., osteogenic, adipogenic, and chondrogenic). They represent a promising tool in tissue engineering for biomedical applications [1,2]. Metabolomics has been increasingly recognized as an important approach to study stem cells (SCs) [3], having the ability to profile intracellular polar and lipidic metabolites (endometabolome), as well as extracellular media (exometabolome). This can contribute to the understanding of the dynamic crosstalk between intra- and extracellular metabolic pathways in several cellular processes, including differentiation [4,5,6]. Most reports to date have addressed the polar metabolites of the endometabolome in differentiating cells [3]; however, increasing evidence suggests that lipids also play a major role in MSCs stemness and lineage commitment [7,8].

Although metabolomics of lipids (or lipidomics) is often associated with mass spectrometry (MS)-based approaches, nuclear magnetic resonance (NMR) spectroscopy is also a valuable tool, particularly in the context of untargeted analysis, as it can detect different lipid families simultaneously, albeit with lesser specificity and sensitivity compared to gas chromatography (GC)-MS or liquid chromatography (LC)-MS [9,10,11]. In fact, NMR and MS strategies have been consistently recognized as complementary for use in one context [12]. The increasing interest in SC lipidomics [8] has resulted in the majority of studies addressing MSCs (mostly using MS [3], along with one report using ^1^H NMR [11]), followed by pluripotent SCs [13,14,15] and other SCs [16,17,18]. The main topics studied have comprised MSC differentiation (into adipogenic [19,20,21,22,23], osteogenic [19,24,25,26,27], or chondrogenic lineages [24,28,29]) and senescence/aging [30,31,32], with most reports employing bone marrow MSCs (BMMSCs) [22,23,25,26,27,28,29,30,32], followed by adipose tissue-derived MSCs (AMSCs) [19,20,24,33,34,35,36,37] and other MSCs [11,31]. Initial differentiation studies using GC-MS found that rat BMMSCs exposed to adipogenic media experienced decreases in saturated/unsaturated fatty acid (FAs) ratios over time, while maintaining a free fatty acid (FFA) profile similar to that of undifferentiated cells [22,23]. Interestingly, differences between differentiated and primary adipocytes could be partially explained by the lack of essential polyunsaturated fatty acids (PUFAs) in culture media [22]. It was also suggested that the presence of the differentiation-inductor dexamethasone (Dexa) in the medium could lead to autophagy and consequent lipid accumulation, without adipogenesis occurring [23]. In addition, a shotgun tandem MS study found that the lipidome is significantly influenced by adipose tissue type and anatomical location. This suggested that different *in vitro* models of adipogenic differentiation may better represent distinct adipose tissue characteristics [21]. For instance, the profiles of hexosylceramides and sphingomyelins (SMs) were found to be important distinguishers of different end adipose tissue types. More recently, hydrophilic interaction LC, in parallel with reversed-phase (HILIC-RP) chromatography, allowed the simultaneous analysis of polar and lipidic extracts of hAMSCs during adipogenic differentiation [20]. In addition, MS imaging techniques have investigated lipid content and spatial distribution in hBMMSCs aggregates (micromasses) in a three-dimensional model of chondrogenesis; this revealed that the distribution of phosphocholine (PC) and diacylglycerols (DGs) seemed to depend on the degree of oxygen availability [29].

The use of metabolites to activate or guide SC differentiation has been outlined in a LC-MS report of the metabolome of adipose perivascular SCs upon hydrogel stiffness-directed chondrogenesis and osteogenesis [24]. Based on the specific depletion of lysophosphatidic acid and cholesterol sulfate (CS), these metabolites were suggested to be inductors of chondrogenesis and osteogenesis, respectively. In subsequent LC-MS studies of hBMMSCs exposed to nanovibrational osteogenic stimulation [26,27], CS was confirmed as a potential osteoinductor, and basal media supplementation with CS did induce the expression of osteogenic markers (although to a lesser extent than traditional osteogenic media) [27]. The osteoinducing properties of other metabolites have also been explored, with fludrocortisone acetate highlighted as a potent and specific osteoinductor (i.e., inducing neither simultaneous adipogenesis nor chondrogenesis). MS studies of differentiating hBMMSCs also showed unique plasma membrane phenotypes for osteogenic and adipogenic lineages [25], with the former leading to plasma membranes enriched in plasmalogens and in longer and more unsaturated FAs (namely, PUFAs), at the expense of monounsaturated FAs (MUFAs). Indeed, media supplementation with docosahexaenoic acid (DHA, an ω-3 PUFA) led to its incorporation into membrane lipids (promoting an osteoblast-like lipidic profile) and more stable membrane microdomains, while increases in cholesterol, MUFAs, and saturated FAs were also reported [25]. These lipid studies have provided an extraordinary amount of intracellular information on SC differentiation, and may be improved by articulation with data from polar endometabolome and exometabolome adaptations, although not yet common, in the same cells [26].

Although less explored than the endometabolome, the study of the SC exometabolome throughout differentiation may provide important information on the cell interactions with their niche, while allowing non-invasive monitoring of SC behaviour [6,38,39,40]. For osteogenesis, initial LC-MS studies addressed extracellular metabolic adaptations of mouse BMMSCs during osteogenic differentiation [38], identifying increases in compounds related to proliferation alone (such as deoxyuridine and orotidine) as well as apparent specific osteogenic markers (e.g., citrate, *cis*-aconitate, succinate, and glycerol). The combination of the endo- and exometabolome during osteogenic differentiation of hBMMSCs further identified lysine degradation and proline metabolism as important ongoing events [6]. The same type of cells, studied by LC-MS in 2D and 3D cultures (the latter involving topographically textured microparticles), revealed that varying levels of osteogenesis were accompanied by distinct metabolic variations in the media [39]. Furthermore, exometabolome changes upon adipogenic and osteogenic differentiations of MSCs demonstrated the strong dependence of media metabolite profiles on the specific lineage commitment [40]. For instance, depletion of several amino acids (e.g., aspartate, histidine, proline, and threonine), palmitate, and pantothenate seemed to be indicative of osteogenic media. These results were integrated with transcriptomic data to develop in silico models describing the expansion, osteogenesis, and adipogenesis of MSCs, potentially revealing the most suitable *in vitro* experimental conditions to optimize lineage commitment.

This work reports, for the first time to our knowledge, an untargeted NMR assessment of intracellular lipidic changes accompanying SC differentiation, that focusses on the osteogenic differentiation of hAMSCs. The results have also been articulated with previously reported changes in the polar endometabolome in the same cells [41], and compared with the compositional changes in the exometabolome during the same time period. This work allows further understanding of the metabolic mechanisms through which osteogenic differentiation occurs, and may allow the identification of osteoinductive metabolites to help regulate cell behaviour towards more efficient outcomes.

## 2. Materials and Methods

### 2.1. Expansion of Human Adipose-Derived Mesenchymal Stem Cells (hAMSCs)

In a humidified incubator (5% CO_2_, 37 °C), hAMSCs (American Type Culture Collection, ATCC PCS-500-011, Manassas, VA, USA) from a single donor were expanded in minimum essential alpha medium (α-MEM, Gibco™ 12000063, Waltham, MA, USA), supplemented with 10% *v/v* heat-inactivated fetal bovine serum (FBS, Gibco™ 10270106) and 1% *v/v* antibiotics (penicillin-streptomycin, Gibco™ 15240062). After reaching ~100% confluence, cells were carefully rinsed with Dulbecco’s Phosphate-Buffered Saline (dPBS, Corning^®^ 55-031-PC, Corning, NY, USA), and passaged using a 0.25% (*v*/*v*) trypsin-EDTA solution (Gibco™ 27250018), at 37 °C for 5 min. The detachment reaction was stopped by adding *α*-MEM.

### 2.2. Osteogenic Differentiation and Sampling

**Figure 1:**
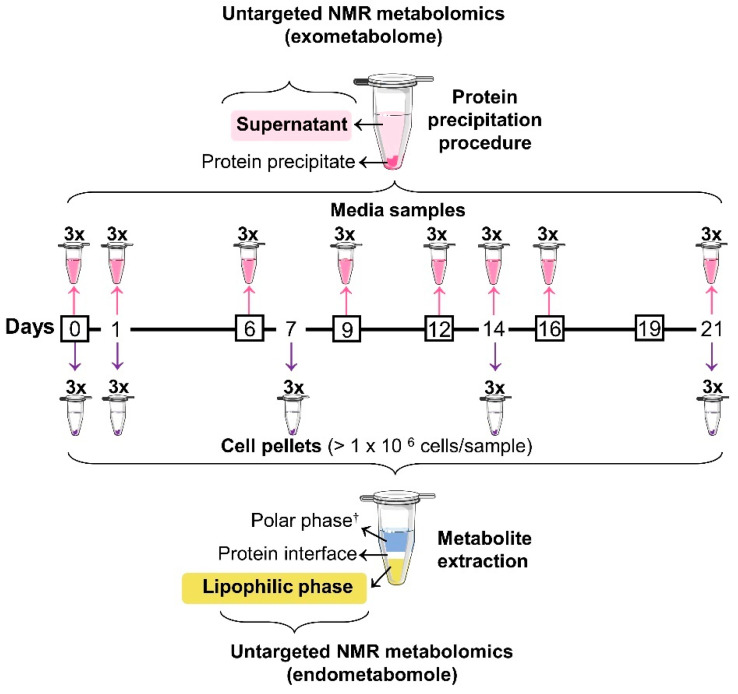
Experimental workflow displaying the osteogenic differentiation timeline (from day 0 to 21). For exometabolome analysis, media samples were collected in triplicate at days 0, 1, 6, 9, 12, 14, 16, and 21 (pink arrows), subjected to a protein-precipitation procedure, and the resulting supernatants were analysed by untargeted ^1^H NMR. Cell samples were collected in triplicate at days 0, 1, 7, 14, and 21 (purple arrows), followed by metabolite extraction and subsequent untargeted ^1^H NMR analysis of the lipidic phase. The corresponding polar endometabolome (^†^) has already been investigated in [41]. Osteogenic medium was exchanged at days 0, 6, 9, 12, 16, and 19 (black boxes). Some elements were adapted from Servier Medical Art and licensed under a Creative Commons Attribution 3.0 Unported (CC BY 3.0) license.

hAMSCs were detached, at passage 7, from the flasks by trypsinization (as mentioned above), counted in a Neubauer chamber and seeded at a density of 0.5 × 10^6^ cells/flask. Cells were maintained under the above-mentioned basal conditions until reaching ~100% confluence, then the culture medium was exchanged and supplemented with osteoinductive factors, specifically 10 mM *β*-glycerophosphate (*β*-GP, Sigma-Aldrich G9422, St. Louis, MO, USA), 50 µg/mL *L*-ascorbic acid (Sigma A0278), and 10 nM Dexa (ACROS Organics™ 230300010, Ho Chi Minh City, Vietnam). The osteogenic media were exchanged twice a week, until day 21 (Figure 1). Quantification of secreted osteocalcin (OCN) and calcium (as described elsewhere [41]) confirmed the occurrence of osteogenic differentiation. hAMSCs conditioned media samples were collected in triplicate at days 1, 6, 9, 12, 14, 16, and 21 after filtration through 40 μm pore strainers. Both fresh (blank) and conditioned osteogenic media samples were processed as described in 2.4. At days 0, 1, 7, 14, and 21 cells were trypsinized, filtered through 100 μm pore strainers, and rinsed twice with PBS, and at least 1 × 10^6^ (range 1–4.5 × 10^6^) cells per pellet were collected for lipidomics. Cell suspensions were centrifuged (300× *g*, 4 °C, 5 min) between each rinsing step.

### 2.3. Lipidic Extraction 

Metabolites were extracted using the methanol-chloroform-water method [42]. Briefly, cell pellets were resuspended in 800 µL of a cold solution of methanol (Honeywell Riedel-de-Haën™ 14262) and Milli-Q water (4:1), allocated to Eppendorf tubes with 150 mg of glass beads (ø = 0.5 mm), and vortexed (2 min, 2500 rpm). Subsequently, 320 µL of cold chloroform (Honeywell Riedel-de-Haën™ 650471) were added, samples were vortexed, followed by the addition of 320 µL of cold chloroform and 288 µL of cold Milli-Q water. After 10 min at −20 °C, samples were centrifuged (10,000× *g*, 15 min, 4 °C). Then, lipophilic and hydrophilic extracts were dried under a nitrogen flow and under vacuum, respectively, and stored at −80 °C. In the present work, the results on lipidic extracts were compared with those reported recently for the corresponding polar extracts [41].

### 2.4. Protein Precipitation in Media Samples

Prior to exometabolome analysis, both blank and conditioned media samples were subjected to a protein-precipitation procedure as previously described [43]. Briefly, 600 µL of 100% methanol at −80 °C were added to microcentrifuge tubes containing 300 µL of each medium sample. After 30 min at −20 °C, samples were centrifuged (13,000× *g*, 20 min), the supernatant was collected, dried under vacuum and stored at −80 °C until NMR analysis.

### 2.5. NMR Spectroscopy 

Dried lipidic extracts were re-suspended in 650 μL of deuterated chloroform (99.8% deuterium, Eurisotop D307F) containing 0.03% tetramethylsilane (TMS) for chemical shift referencing. After vortex homogenisation, 550 μL of solution were transferred to 5 mm NMR tubes. For exometabolome analysis, dried media samples were resuspended in 700 µL of 100 mM phosphate buffer at pH 7.4, previously prepared in D_2_O (99.9% deuterium, Eurisotop D216) and 0.1 mM 3-(trimethylsilyl)-propionic-2,2,3,3-d4 acid (TSP in D_2_O, Sigma-Aldrich 293040), for chemical shift referencing. After a 5 min centrifugation (13,000× *g*) to remove additional proteins that precipitated during storage at −80 °C, 550 μL of the supernatant were transferred to 5 mm NMR tubes. Standard 1D ^1^H NMR spectra were acquired with a standard 90° pulse sequence (*zg* pulse sequence for lipidic extracts) or with water presaturation (*noesypr1d* pulse sequence for media samples) on a Bruker Avance III spectrometer operating at 500.13 MHz (at 298 K). A total of 512 scans (lipidome) or 256 scans (exometabolome) were collected into 32,768 data points using a spectral width of 7002.801 Hz, an acquisition time of 2.3 s and a relaxation delay (d1) of 4 s. Prior to Fourier transformation, each FID (free induction decay) was zero-filled to 65,536 points (lipidome) or 131,072 points (exometabolome) and multiplied by a 0.3 Hz exponential line-broadening function. Subsequently, spectra were manually phased, baseline corrected, and chemical shifts referenced internally to TMS (lipidic extracts) and TSP (media samples) at *δ* = 0.00 ppm. Peak assignments were based on 2D ^1^H−^1^H total correlation (TOCSY) and 2D ^1^H−^13^C heteronuclear single quantum correlation (HSQC) spectra analysis, the literature [44,45,46,47,48], spiking experiments, and spectral databases, such as the human metabolome database (HMDB) [49] and Chenomx NMR Suite (Chenomx, Edmonton, AB, Canada).

### 2.6. Data Analysis and Statistics

Prior to multivariate analysis (MVA) of the lipidomic data, ^1^H NMR spectra were aligned using the recursive segment-wise peak alignment method [50] (Matlab R2014a, The MathWorks Inc., Natick, MA, USA) and normalised to total spectral area in order to minimise chemical shift variations and sample concentration differences, respectively. Multivariate analysis [51] was applied to the full resolution ^1^H NMR spectra using SIMCA-P 11.5 (Umetrics, Umeå, Sweden), excluding the regions of residual water (*δ* 1.8–1.4), a broad unassigned signal present in only one sample (*δ* 4.8–4.6) and chloroform (*δ* 7.5–7.0). Principal component analysis (PCA) and partial least squares discriminant analysis (PLS-DA) were performed after pareto and unit variance (UV) scalings of the spectra, respectively. The loadings plot (Matlab R2014a) corresponding to the best performing PLS-DA model (days 0 and 1 vs. 7, 14, and 21) was obtained by multiplying loading weights by the standard deviation of each variable, and the colour scale reflected variable importance to the projection (VIP). For univariate analysis, all peaks from the original spectra were integrated using Amix 3.9.15 (Bruker BioSpin, Rheinstetten, Germany) and normalised to total spectral area. Using Python 3.9, Wilcoxon rank-sum nonparametric tests [52] were applied to the original normalized integrals to determine statistically significant differences (*p*-values < 0.05). For the most relevant lipidic variations (i.e., statistically significant differences confirmed by visual inspection), the effect size (ES) values and corresponding errors were calculated [53]. For multiple testing, these changes were also considered for the Benjamini–Hochberg false discovery rate (FDR) [54] correction, and the resulting FDR adjusted *p*-values < 0.05 were considered significant. In addition, average FA chain length, unsaturation index, and polyunsaturation index were carried out as described previously [55].

As described above for the lipidome, media ^1^H NMR spectra were aligned and normalised. Importantly, prior to MVA and to create the trajectory line graphs for the most relevant exometabolome variations related only to the osteogenic process, a correction was needed to compensate for the effect of media exchanges (at days 0, 6, 9, 12, 16, and 19, Figure 1). Therefore, after the first media renovation (at day 6) the following equations were applied to each of the three samples from day i, allowing for the continuous process of osteogenesis to be more easily analysed and interpreted.

(1) $$D_{i}={\bar{D}}_{i-1}\;+({\mathrm{OD}}_{i}-\bar{B})$$

(2) $$D_{i}={\bar{D}}_{i-1}+({\mathrm{OD}}_{i}-{\bar{\mathrm{OD}}}_{i-1})\;$$

where D_i_ is the corrected normalized integral of day i; $\bar{D}$_i−1_ is the mean of the normalized integrals of the previous collection day (when the previous collection day was also corrected, $\bar{D}$_i−1_ should consider the corrected normalized integrals); OD_i_ is the original normalized integral of day i; $\bar{B}$ is the mean of the normalized integrals of blank media samples; and $\bar{\mathrm{OD}}$_i−1_ is the mean of the original normalized integrals of the previous day. Importantly, Equation (1) should be applied when media exchange occurs between consecutive timepoints (or collection days), and Equation (2) should be used when there was no media exchange between consecutive timepoints (in our case, this only applies in relation to day 16). Pareto scaling was applied before PCA analysis. Wilcoxon rank-sum nonparametric tests [52] were applied to the original normalized integrals (not corrected), and all statistically significant differences (*p*-values < 0.05) were also confirmed by visual inspection.

## 3. Results

### 3.1. hAMSC Lipophilic Endometabolome Changes

**Figure 2:**
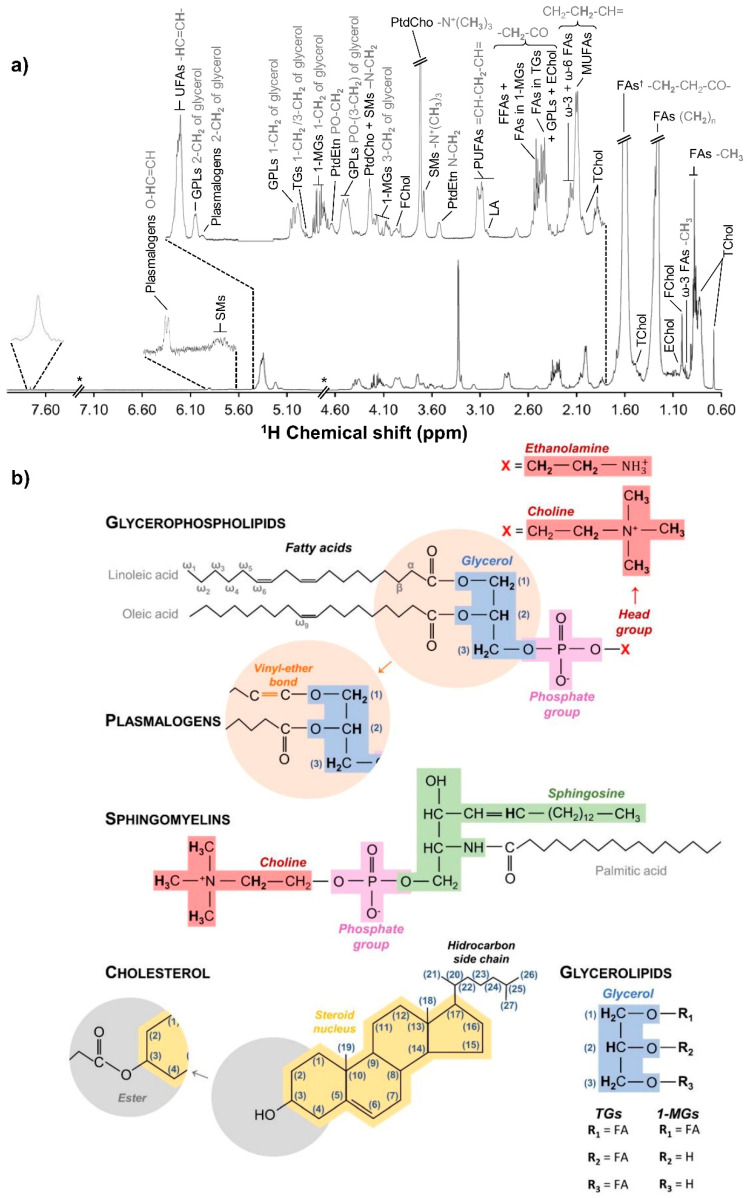
Lipidic profile of human adipose-derived mesenchymal stem cells (hAMSC). (**a**) Typical ^1^H NMR spectra (500 MHz) of lipidic extracts from undifferentiated hAMSC prior to osteogenic medium exposure. *: exclusion of a broad unassigned signal (*δ* 4.8–4.6) and chloroform (*δ* 7.5–7.0) resonances; ^†^: due to overlapping with residual water, the peak assigned to -CH_2_-CH_2_-CO- of FAs (*δ* 1.8–1.4) was not considered for any statistical analysis. (**b**) Chemical structures of representative lipid species from the main families here identified, namely glycerophospholipids (including plasmalogens), sphingomyelins, cholesterol (free and esterified), and glycerolipids (TGs and 1-MGs). Protons in bold correspond to those assigned in the NMR spectra. Supplemental Table S1 shows the complete list of assigned signals. 1-MGs, 1-monoacylglicerides; EChol, esterified cholesterol; FAs, fatty acids; FChol, free cholesterol; FFAs, free fatty acids; GPLs, glycerophospholipids; MUFAs, monounsaturated fatty acids; PtdCho, phosphatidylcholine; PtdEtn, phosphatidylethanolamine; PUFAs, polyunsaturated fatty acids; SMs, sphingomyelins; TChol, total cholesterol; TGs, triacylglycerides; and UFAs, unsaturated fatty acids.

Figure 2a shows a representative ^1^H NMR spectrum of lipophilic extracts of undifferentiated hAMSCs (day 0) and Table S1 lists all peak assignments achieved, corresponding to several lipid families (Figure 2b). Specific resonances could be identified for free and esterified cholesterol, MUFAs, PUFAs (both ω-3 and ω-6 PUFAs, including linoleic acid (LA)), several phospholipids (namely, phosphatidylcholine (PtdCho), phosphatidylethanolamine (PtdEtn), plasmalogens and sphingomyelins (SMs)), 1-monoacylglicerides (1-MGs), and triacylglycerides (TGs).

**Figure 3:**
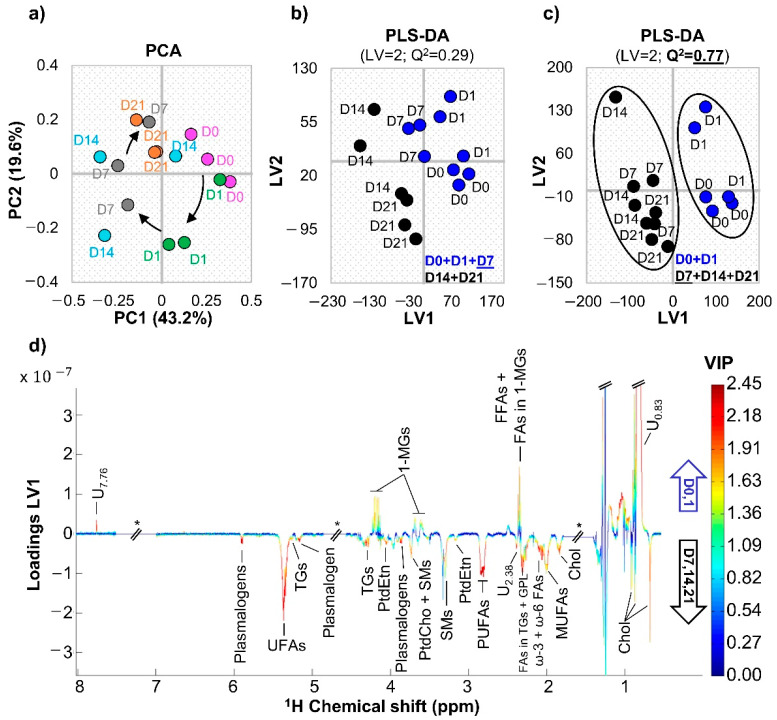
Multivariate statistical analysis considering full resolution ^1^H NMR spectra of lipidic extracts from hAMSC throughout 21 days of osteogenic differentiation. (**a**) Scores scatter plots for principal component analysis (PCA) containing days 0 (pink), 1 (green), 7 (grey), 14 (blue), and 21 (orange), all in triplicate. (**b**,**c**) Scores scatter plots for partial least-squares discriminant analysis (PLS-DA) comparing the initial days of osteoinduction (blue) with the later days (black), considering day 7 as an (**b**) initial or (**c**) later day. (**d**) LV1 loadings plot (coloured according to variable importance to the projection, VIP) related to the PLS-DA model present in (**c**). Abbreviations as defined in Figure 2. Di: day i; LV: latent variables; Q^2^: predictive power; and U*_δ_*: unassigned signal at chemical shift *δ*.

**Figure 4:**
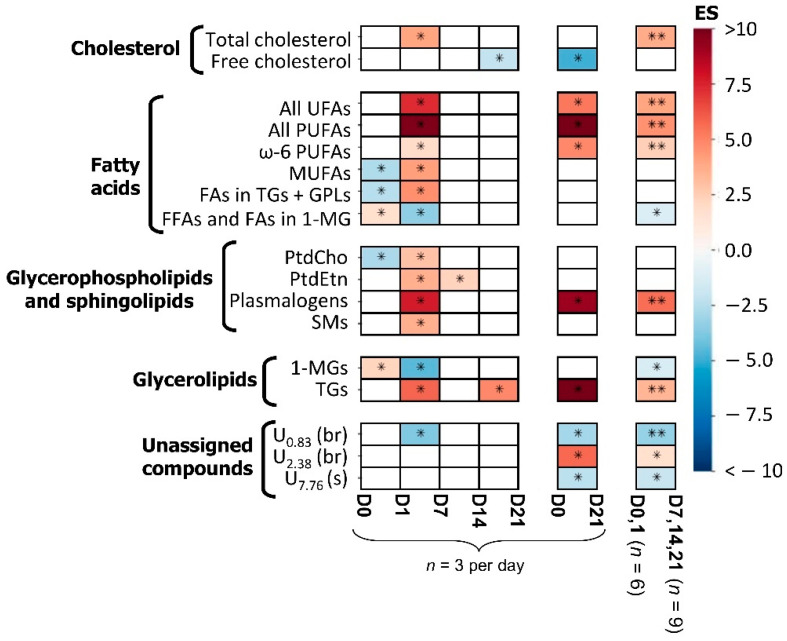
Heatmap showing statistically significant lipidic variations observed during osteogenic differentiation of hAMSCs, selected by spectra visual assessment and coloured according to the effect size (ES) values, from minimum (dark blue) to maximum (dark red). Rows correspond to lipidic species/classes and columns allow for comparisons between consecutive analysed timepoints between extreme days (days 0 vs. 21) and between classes (days 0 and 1 vs. 7, 14, and 21). Abbreviations as defined in Figure 2. Di: day i; U*_δ_*: unassigned signal at chemical shift *δ*; *: Wilcoxon Rank-sum *p*-value < 0.05; and **: Wilcoxon Rank-sum *p*-value < 0.01.

The PCA scores plot of the ^1^H NMR spectra of all samples collected throughout osteogenic differentiation (Figure 3a) indicated a progression from day 0 to day 7, which overlaps with day 14 samples, and finally to day 21. Notably, days 7 and 14 of osteogenic differentiation show larger sample dispersion, suggesting that the high eventful nature of such stages leads to possible small experimental variations (between replica) impacting importantly on lipidic profiles. Interestingly, the first and last days of differentiation seem positioned close together, which suggests that some features return to initial levels (this was also seen for the polar metabolome in the same cells [41]). Pairwise PLS-DA models obtained for the lipophilic extracts performed weakly (Q^2^ = 0.29) when day 7 was grouped with day 0 and day 1 (Figure 3b), whereas a robust model was obtained (Q^2^ = 0.77) when day 7 was included in the same class as days 14 and 21 (Figure 3c). This indicates that the profile of day 7 samples is closer to those of days 14 and 21, suggesting that the main biochemical changes in lipophilic metabolites take place before day 7. Indeed, PLS-DA loading analysis (Figure 3d) showed that the main changes occurring up to day 7 affected total cholesterol, unsaturated FAs (UFAs), MUFAs, PUFAs (specifically including ω-6 PUFAs), several phospholipids, TGs, 1-MGs, and unassigned resonances at δ 0.83, δ 2.38, and δ 7.76. Spectral integration confirmed that the larger number and magnitude of spectral changes (as viewed through effect size) indeed took place from day 1 to day 7 (Figure 4 and Table 1 and Table S2): increases in total cholesterol, MUFAs, PUFAs, and phospholipids (PtdCho, PtdEtn, SMs, and plasmalogens) and decreased 1-MGs. However, a few changes also occurred from day 0 to day 1 (decrease in MUFAs, FAs in TGs + GPLs, and PtdCho, and increase in 1-MGs), and after day 7 (increased PtdEtn in day 14, and decreased free cholesterol and increased TGs in day 21). Comparison of earlier (days 0 and 1) and later (days 7, 14, and 21) stages showed persistently high total cholesterol, PUFAs, plasmalogens, and TGs, and lower 1-MGs (Figure 4), while MUFAs and phospholipids (PLs) levels (except for plasmalogens) tend to recover initial values. All significant changes were confirmed by visual inspection of the NMR spectra as illustrated by examples in Figure S1.

**Figure 5:**
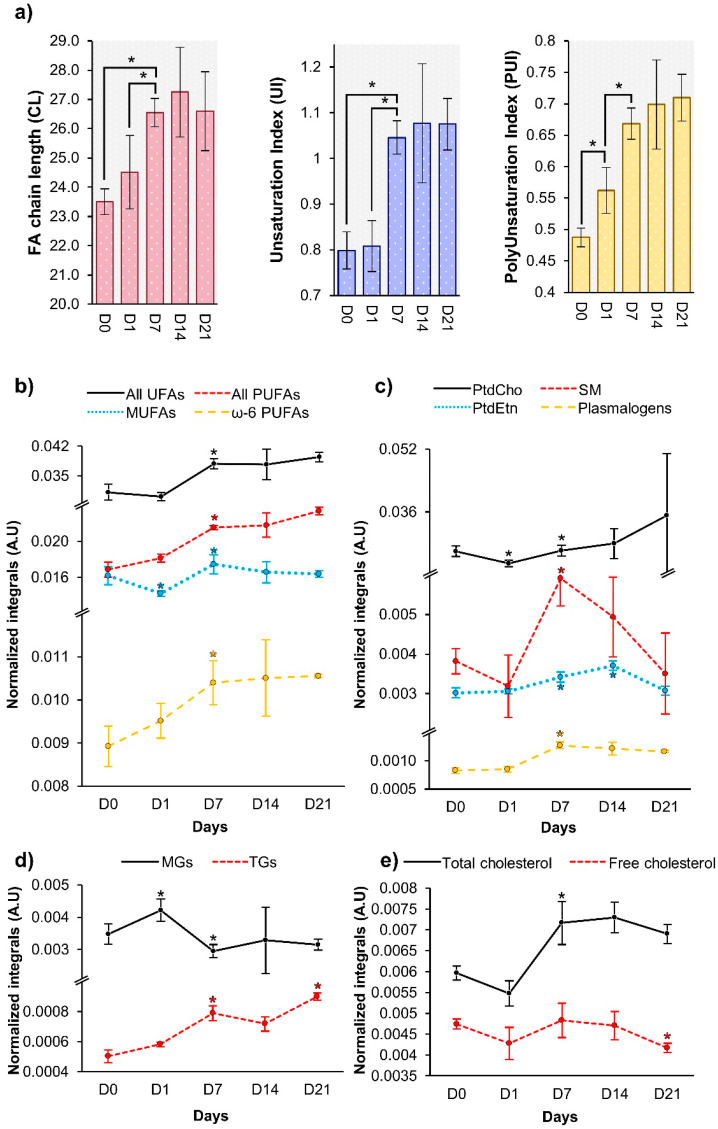
Average lipid characteristics (**a**) and level fluctuations (**b**–**e**) of lipid species/classes selected in Figure 4. (**a**) Average fatty acid chain length, unsaturation index and polyunsaturation index were calculated according to [55]. Normalized integrals of lipids during the 21 days of osteogenic differentiation, including (**b**) fatty acids, (**c**) glycerophospholipids and sphingomyelins, (**d**) cholesterol, and (**e**) glycerolipids. Abbreviations as defined in Figure 2. Di: day i; *: Wilcoxon Rank-sum *p*-value < 0.05 (specifically in (**b**–**e**) graphs, each day was compared to the previous timepoint).

In addition, average FA chain length and degree of polyunsaturation increased up to day 7 (Figure 5a). The average degree of polyunsaturation is indeed correlated with a gradual increase in PUFAs (including ω-6 PUFAs) from day 0 to day 7, with MUFAs increasing between days 1 and 7 (Figure 5b). After day 7, no significant changes were observed in FA moieties (Figure 5a,b). The newly synthesized FAs may be incorporated into PLs (Figure 5c) and glycerolipids (Figure 5d), all PLs detected showing significant increases from day 1 to day 7, with PtdEtn increasing further up to day 14. Increasing TG levels seemed to mirror decreasing MGs, suggesting a conversion of MGs into TGs (Figure 5d). Finally, total cholesterol levels were confirmed to increase significantly from day 1 to day 7 (Figure 5e), reflecting a small increasing tendency in predominating free cholesterol, and suggesting that esterified cholesterol may be increasing too (although only a qualitative increase was observed in the weak resonance at δ 1.02).

### 3.2. hAMSC Exometabolome Changes

**Figure 6:**
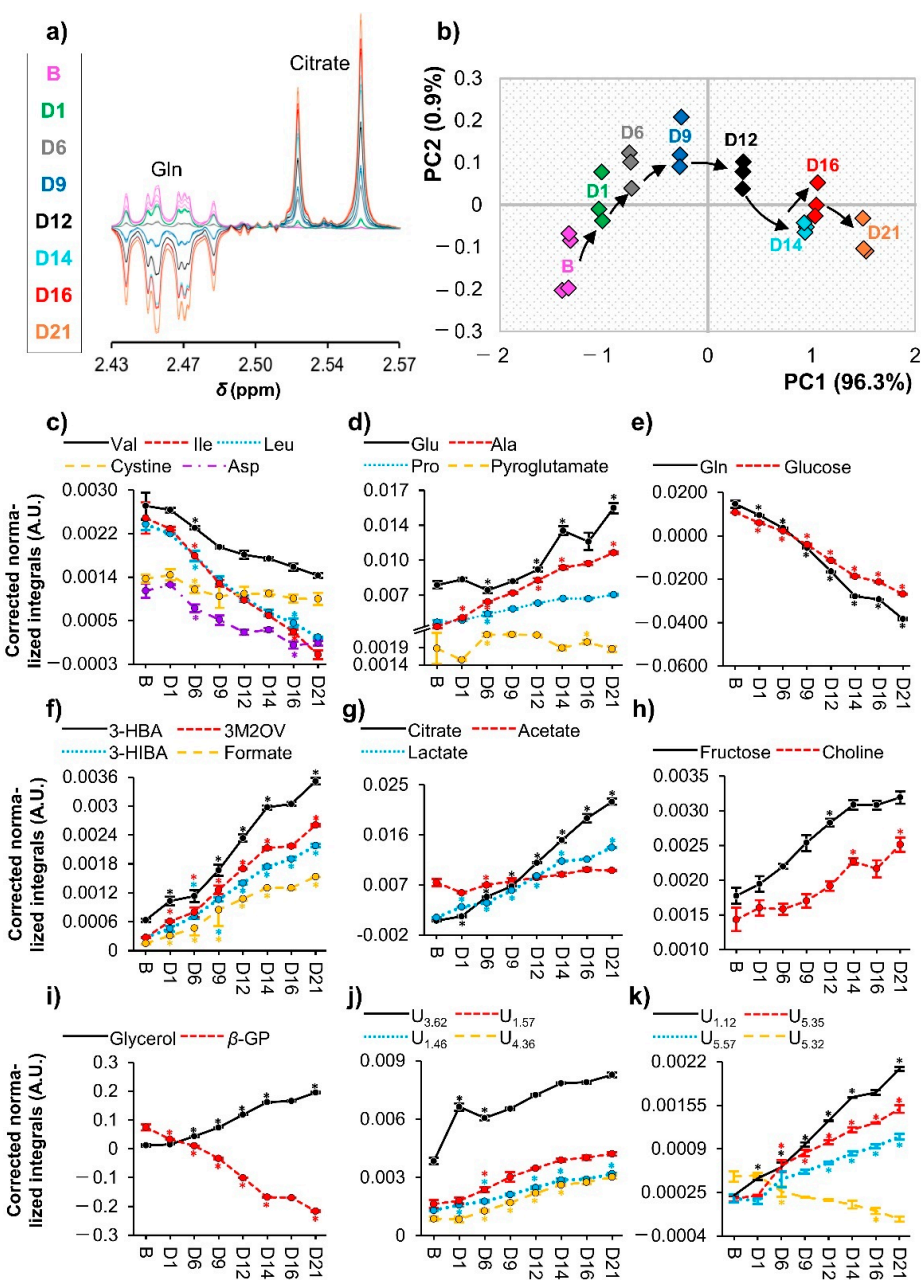
Main exometabolome variations throughout osteogenic differentiation. (**a**) Representative NMR spectral region corrected to compensate for the effect of media exchanges; (**b**) Scores scatter plots for principal component analysis (PCA) comparing osteogenic medium blank samples (n = 4) and samples collected throughout differentiation (at days 1, 6, 9, 12, 14, 16, and 21, each in triplicate); (**c**–**k**) line graphs display the corrected normalized integrals over time for (**c**,**d**) some amino acids and derivatives. (**e**) glutamine and glucose, (**f**,**g**) organic acids, (**h**,**i**) other identified metabolites and (**j**,**k**) unassigned resonances. Three-letter code used for amino acids. 3-HBA, 3-hydroxybutyrate; 3-HIBA, 3-hydroxyisobutyrate; 3M2OV, 3-methyl-2-oxovalerate; and *β*-GP, *β*-glycerophosphate. B: blank (fresh) osteogenic medium samples; Di: day i; U*_δ_*: unassigned signal at chemical shift *δ*; and *: Wilcoxon Rank-sum test p-value < 0.05 compared to the previous timepoint and represented in the same colour as the corresponding line (shown only in cases where spectral confirmation was observed).

The detailed peak assignment of the ^1^H NMR spectra for cell media across osteogenesis (see example in Figure S2) enabled the identification of 38 metabolites (Table S3), along with the osteogenic supplements ascorbate and *β*-GP (Dexa was not detected, most probably due to its low concentration, *ca.* 10 nM in osteogenic blank media), and DMA (found present in the dexamethasone supplement). All compounds are listed in Table S3, including several amino acids (19 in total) and derivatives (creatine, cystine, and pyroglutamate), organic acids (3-hydroxybutyrate (3-HBA), 3-hydroxyisobutyrate (3-HIBA), and 3-methyl-2-oxovalerate (3M2OV), acetate, citrate, formate, lactate, pyruvate, and succinate), and other compounds (acetone, choline, fructose, glucose, glycerol, methylguanidine, and *myo*-inositol). Upon correction for media exchanges after day 6 (as described in the experimental section), which led to metabolite variations illustrated such as in Figure 6a), the PCA of the resulting corrected media spectra (Figure 6b) showed a clear trajectory up to day 14 (with a similar profile to day 16 media) and then to day 21. As illustrated in Figure 6a, media exchange correction leads to negative integral values, reflecting lower amounts compared to those in the original blank solution (due to cellular uptake).

Many of the amino acids or derivatives observed to vary (the three branched chain amino acids (BCAAs), aspartate, glutamine, and cysteine dimer (cystine)) showed marked decreases from day 0, although only becoming statistically significant from day 1 to day 6 (Figure 6c,e). On the other hand, alanine, glutamate, and proline were excreted, with pyroglutamate maintaining higher values from day 6 to day 12 (Figure 6d). All the varying acids (3-HBA, 3-HIBA, 3M2OV, acetate, citrate, formate, and lactate) steadily increased in cell media throughout osteogenic differentiation, with acetate tending to level out more, compared to other acids (Figure 6f,g). Media glucose was heavily consumed (Figure 6e), whereas fructose was clearly excreted, along with an increase in extra cellular free choline (Figure 6h). The osteogenic inductor *β*-GP rapidly decreased in cell media, whereas glycerol levels increased (Figure 6i). In addition, many still unassigned resonances (Figure 6j,k) showed clear trajectories too, and work is underway to determine these assignments.

## 4. Discussion

### 4.1. The Dynamics of hAMSC Endometabolome during Osteogenic Differentiation

**Figure 7:**
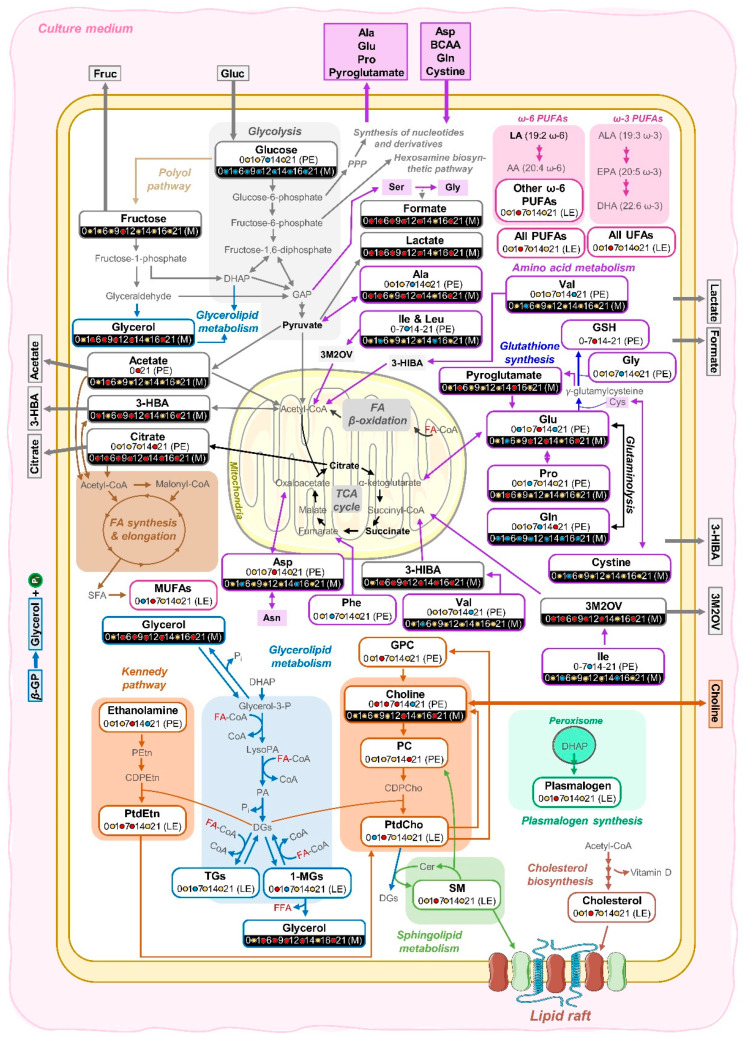
Schematic illustration of the main intracellular and extracellular metabolic variations detected during the osteoinduction of hAMSCs. Metabolites indicated in bold correspond to those detected by NMR. Metabolites that fluctuate over time show a schematic scale underneath (in white background for lipid and polar extracts (LE and PE, respectively), and in black background for media samples (M), where unchanged, increased, and decreased levels are shown in yellow, red, and blue circles, respectively. It should be noted that information on polar extracts refers to previously reported data [41]. Arrows crossing the cell membrane represent metabolites exchanged between intra- and extracellular environments. Abbreviations: 3-letter code used for amino acids; 1-MG, 1-monoacylgliceride; 3-HBA, 3-hydroxybutyrate; 3-HIBA, 3-hydroxyisobutyrate; *β*-GP, *β*-glycerophosphate; AA, arachidonic acid; ALA, linolenic acid; BCAA, branched chain amino acids; CDPCho, Cytidine diphosphocholine; CDPEtn, cytidine diphosphoethanolamine; Cer, ceramide; CoA, coenzyme A; DGs, diacylglicerides; DHA, docosahexaenoic acid; DHAP, dihydroxyacetone phosphate; EChol, esterified cholesterol; EPA, eicosapentaenoic acid; FA-CoA, fatty acyl coenzyme A; FAs, fatty acids; FChol, free cholesterol; FFAs, free fatty acids; Glycerol-3-P, glycerol-3-phosphate; GPC, glycerophosphocholine; GSH, glutathione (reduced); LA, linoleic acid; LysoPA, lysophosphatidic acid; MUFAs, monounsaturated fatty acids; PA, phosphatidic acid; PC, phosphocholine; PEtn, phosphoethanolamine; PPP, pentose phosphate pathway; PtdCho, phosphatidylcholine; PtdEtn, phosphatidylethanolamine; PUFAs, polyunsaturated fatty acids; SFA, saturated fatty acis; SM, sphingomyelin; TCA, tricarboxylic acid; TGs, triacylglyceride; and UFA, unsaturated fatty acids. Some elements were adapted from Servier Medical Art and licensed under a Creative Commons Attribution 3.0 Unported (CC BY 3.0) license.

The results and possible explanations for both endometabolome and exometabolome dynamic adaptations are shown in Figure 7. Similarly to that reported for the polar endometabolome for the same hAMSCs [41], lipid metabolism adaptations seem to subdivide osteogenic differentiation into different stages in which distinct events seem to occur. Between days 0 and 1 (Figure 4 and Figure 5b–d), 1-MGs increase, possibly at the expense of FAs present in TGs and/or GPLs, also accompanied by a decrease in PtdCho and MUFAs. As these variations are subsequently inverted between days 1 and 7, these early variations seem to consist of preparation steps for the more eventful stage that follows. 

Interestingly, a similar behaviour was seen for intracellular amino acids [41], with a slight increase at days 0–1, only to markedly decrease thereafter, probably to support early protein synthesis. At the same time, polar extracts indicated a significant investment of ATP and phosphocreatine (PCr) for energy and inorganic phosphate (P_i_) production (the latter destined to incorporate in extracellular hydroxyapatite) [41]. Later, between days 1 and 7, 1-MGs are used up, probably to support TG increases (Figure 5d), for energy storage in lipid droplets which have been demonstrated to accumulate throughout MSC osteogenic differentiation [56]. Although high levels of TG accumulation are usually related to adipogenic differentiation, we believe that parallel adipogenesis should be residual, given the standard osteogenic conditions employed (although specific biomarkers, not measured here, would be useful to ascertain this). Concomitantly to the 1-MGs and TGs variations, the significant increase in the levels of unsaturated FAs (particularly MUFAs and PUFAs, including ω-6 PUFAs) up to day 7 (Figure 4 and Figure 5a,b) may reflect a protective mechanism through which saturated FAs (that can induce cytotoxicity in MSCs and osteoblasts [57]) are, at least partially, replaced by UFAs. It is important to note that, in addition to intracellular lipidic synthesis, FBS is the main external source of lipids [58] (although containing low lipid content, especially regarding PUFAs), also claimed to alter *in vitro* FA composition of MSCs, compared to *in vivo* [31]. Furthermore, ω-6 PUFAs are clearly seen to increase, whereas ω-3 PUFAs do not change significantly; it seems, therefore, that the former are aiding osteogenic differentiation, in apparent contradiction of previous reports of their inhibitory effect when used as media supplements (while ω-3 have a promoting role) [25,59,60]. However, such reports may be dose-dependent and/or even reflect different mechanisms depending on the original location of the FA (i.e., in endo- or exometabolomes). Several PLs (PtdCho, PtdEtn, plasmalogens, and SMs) also increase between days 1 and 7 (Figure 4 and Figure 5c), and this is accompanied by increases in PL precursors GPC and choline in polar extracts [41], probably to support PL synthesis (Figure 7). This increase is most likely related to membrane remodelling, possibly through the incorporation of higher content of unsaturated FAs, thus increasing membrane fluidity. Indeed, membrane fluidity has been shown to be higher in cells undergoing osteogenic differentiation than in proliferating cells [61], particularly after day 7. The average FA chain length (Figure 5a) also increased significantly at this point (between days 1 and 7), although it remains unclear if this reflects FAs incorporating newly synthesized TGs, and/or the new membrane PLs. The latter explanation would be expected to counteract the effect of increasing unsaturated/polyunsaturated FAs on membrane fluidity. However, a study focused on hBMMSC membranes alone, during osteogenic differentiation, did report enhanced levels of longer and more unsaturated FAs [25]. The increase in total cholesterol confirmed previous observations [62,63], but the direct interpretation of this is rather complex as cholesterol has multiple possible roles, from impacting on cell fluidity [61], to having an osteogenic-promoter role (e.g., as demonstrated for the CS form [24,27]), with its esterified forms (here seen to qualitatively increase slightly) specifically known to stimulate MSC osteogenic differentiation [64]. In addition, an interesting recent report [65] has demonstrated distinct roles for cholesterol depending on its endogenous or exogeneous origin, resulting in osteogenesis stimulation and inhibition, respectively. Cholesterol is also an important component of lipid rafts (Figure 7), along with SMs and plasmalogens [66], with the exact nature of these formations known to play an important role in the equilibrium between stimulus and inhibition of osteogenesis [67]. Furthermore, cholesterol synthesis is related to vitamin D metabolism, known for its important role in osteogenesis [68]. During the same period (days 1–7), amino acids are being extensively used for protein synthesis [41], along with protein *O*-glycosylation reactions and the activation of anti-oxidative mechanisms (through increased intracellular glutathione, GSH). The latter mechanisms may also relate to the plasmalogen increase, as these species act through the vinyl ether bonds (Figure 2b) to reduce the levels of reactive oxygen species (ROS) [69]. After day 7, UFA synthesis seems to stabilize (Figure 4 and Figure 5b), while PLs tend to return to initial levels, except for plasmalogens that (together with GSH [41]) remain elevated (Figure 4), probably to sustain anti-oxidative mechanisms. The decrease in PLs may indicate a slowing down of cell proliferation rates after day 7 as suggested previously [41], although high glycolytic and glutaminolytic activities were evident until day 14 (Figure 7), as were ROS scavenging mechanisms. However, such high metabolic activity does not seem to impact significantly on lipid metabolism, as only small changes are noted after day 7, namely in PtdEtn (reported to promote osteogenesis [70] and consistent with decreasing ethanolamine in polar extracts [41]), free cholesterol, and TGs.

Overall, the lipidic endometabolome of differentiated hAMSCs seems to be characterized by MUFAs and PL levels approaching initial values (except for plasmalogens), while differences remain regarding increased total cholesterol, PUFAs, TGs, and plasmalogens, and decreased 1-MGs. Some polar metabolites have also been seen to return to initial levels in the same cells [41], suggesting that parts of cellular adaptations are temporarily in place to ensure osteogenic commitment.

### 4.2. Articulation between the Endo- and Exometabolomes of hAMSCs during Osteogenic Differentiation

Media levels of amino acids and derivatives are expected to change significantly during differentiation [6]. This work shows the preferential use of the three BCAAs (leucine, isoleucine, and valine), aspartate, and glutamine, together with the cysteine dimer (cystine), throughout the whole osteogenic process (Figure 6c,e). These amino acids may be playing a specific role in intracellular protein synthesis, including that of osteogenic markers osteocalcin and osteopontin [71], which contain aspartate and glutamate (the latter also provided through active glutaminolysis [41]). Except for cystine, all the imported amino acids are anaplerotic [72], hence probably feeding the TCA cycle (Figure 7) for energy production and formation of citrate, subsequently excreted into the media for extracellular mediation of hydroxyapatite formation [73]. The decrease in extracellular cystine may be explained by its known role to penetrate cells, in exchange for glutamate (mediated by cystine-glutamate antiporters), in order to provide enough cysteine for GSH maintenance/modulation for oxidative stress protection [74]. In turn, excreted glutamate plays a powerful role as a mineralization mediator [75,76], similar to citrate.

In addition to amino acids, glucose is consumed promptly (Figure 6e) for sustaining the increased glycolytic activity based on the polar endometabolome [41], whereas the increase in lactate observed in media suggests that cell glycolysis may, at least partially, lead to active fermentation (Figure 7), similar to the Warburg effect in tumour cells as reported before for proliferating MSCs [77]. To our knowledge, the equilibrium between oxidative phosphorylation (OxPhos) and lactate formation in the present context is yet to be clarified and would benefit from further investigation [77,78,79]. Similarly, no clear explanation may be advanced, at this stage, for the export of fructose, although it is known that fructose plays an adipogenic induction role [80,81]. We hypothethise that the fact that cells undergoing osteogenic differentiation are significantly exporting this sugar may mean that co-adipogenesis may be limited. In addition, the role of fructose in bone tissue formation and characteristics has been discussed [82], suggesting that it can act as a modulator of the adipo-osteogenic commitment of MSCs.

Besides lactate and citrate, 3-HBA, 3-HIBA, 3M2OV, acetate, and formate also showed a steady increase in cell media (Figure 6f,g). The accumulation of the ketone body 3-HBA in media may be indicative of ongoing intracellular FA *β*-oxidation (an essential energy source for the osteogenic process [83,84,85]). However, 3-HBA was not detected in the endometabolome [41], which may mean that it is rapidly excreted (Figure 7), perhaps to act as a signalling metabolite *via* extracellular receptors [86]. Notably, it has been shown that *in vitro* and *in vivo* 3-HBA supplementation results in enhanced osteogenic differentiation [87]. In addition, both 3-HIBA and 3M2OV are products of isoleucine and valine breakdown [88], which are taken up by the cells at the start of osteogenesis. This may indicate incomplete use of the BCAAs isoleucine and valine as anaplerotic substrates (Figure 7), perhaps to help modulate TCA activity, and hence OxPhos extension. Acetate production is important in maintaining acetyl-CoA pools (Figure 7) and could, at least partially, result from ROS-mediated pyruvate decarboxylation (especially during activated glucose metabolism) [89], also supporting the already discussed antioxidant capacity throughout osteogenic differentiation. Although formate accumulation has not, to the best of our knowledge, been linked to MSCs differentiation, studies on other mammalian cells have revealed that mitochondrial energy generation from serine one-carbon catabolism is a major source of formate [90], with alternative sources also being suggested (such as cholesterol synthesis) [91]. *β*-GP is a standard extracellular source of inorganic phosphate in osteogenic media (Figure 7) [92], mainly through the action of several upregulated phosphatases; thus, the mirroring trajectory of *β*-GP and glycerol was expected, and extracellular accumulation of glycerol during osteogenesis of MSCs has indeed been previously reported [6,38]. As an interesting note, the use of *β*-GP as a phosphate source, instead of the direct addition of inorganic phosphate, has been recognized as a debatable issue [93].

In addition, extra- and intracellular choline levels are seen to increase (Figure 6f) until day 14 [41]. We first hypothesized that, as intracellular GPC and PC did not decrease, the source of excess choline inside the cell might be PtdCho and SM hydrolysis from day 0 to day 1 of osteogenic differentiation (Figure 5c). However, an additional explanation for high choline levels, both outside and inside the cell, may reside in the expected upregulation of phosphoethanolamine/phosphocholine phosphatase (PHOSPHO1) during bone mineralization [94,95]. The PHOSPHO1 enzyme is believed to be released from cells into matrix vesicles, where it generates P_i_ from PC (for hydroxyapatite deposition). We suggest that vesicle rupture will contribute to extracellular choline accumulation, possibly also leading to higher intracellular choline levels to sustain PLs increase/stabilization later in the osteogenic process (this is expressed by a double arrow in Figure 7).

## 5. Conclusions

This paper describes, for the first time to our knowledge, a lipidomic and exometabolome characterization of the osteogenic differentiation of hAMSCs in 2D cultures, through NMR metabolomics. Our results describe the dynamic time-course evolution of cholesterol, MUFAs, and PUFAs (including ω-6 and ω-3 fatty acids), several PLs (PtdCho, PtdEtn, SMs, and plasmalogens), 1-MGs, and TGs. Most intracellular lipid changes occur between days 1 and 7 of the osteogenic process, although some preparation steps occur beforehand (apparently concerted with specific initial changes in the polar endometabolome of the same cells). Longer and more unsaturated fatty acids increase before day 7, along with PLs, probably for necessary plasma membrane fluidity adaptations. On the other hand, a tendency for lipidome stabilization is observed after day 7, whereas concomitant high metabolic activity affecting polar metabolites was previously reported, with enhanced energetic metabolism in tandem with a Warburg-like effect. The overall (lipidic and polar) dynamic endometabolome changes have been articulated with simultaneous changes observed in media metabolites, enabling important correlations to be established and supporting hypotheses related to the dynamics of membrane remodelling, anti-oxidative mechanisms, phosphate production, protein synthesis, and energy metabolism in hAMSC osteogenic differentiation. Furthermore, in spite of the limitations of this work (namely in relation to addressing a single donor, not accounting for cell ageing adaptations and not including potential co-adipogenesis markers), we believe that the reported articulation between *in vitro* endo- and exometabolome adaptations paves the way for the identification of potential inductive metabolites for optimized osteogenic protocols. This may be of use, not only to approach aspects of 3D cultures and *in vivo* osteogenesis, but also to identify key metabolic traits that may enable *in vitro* osteogenic differentiation of hAMSCs to be optimized, for example for the production of high numbers of pure hAMSCs-derived osteoblasts to be used in tissue engineering constructs.

## Figures and Tables

**Table 1:** Main lipidic variation between the initial days (0 and 1) and later days (7, 14, and 21) of osteogenic differentiation of hAMSCs. All differences presented were confirmed by visual inspection of the spectra and are statistically significant (Wilcoxon Rank-sum test *p*-values < 0.05) [52]. Effect size (ES) values and corresponding errors were calculated as described in reference [53] (level increases and decreases in the later days are represented by positive and negative ES values, respectively). Benjamini-Hochberg false discovery rate (FDR) [54] correction was applied for multiple testing, and all differences showed FDR adjusted *p*-values < 0.05. Abbreviations as defined in Table S1. D_i_: day i; U*_δ_*: unassigned signal at chemical shift *δ*. ^a^ peak used for integration (part of the spin system).

Metabolite	*δ* ^1^H (Multiplicity) ^a^	D_0_ + D_1_ vs. D_7_ + D_14_ + D_21_		
		Effect Size (ES Error %)	*p*-Values	FDR Adjusted *p*-Values
**Cholesterol**				
**Total cholesterol**	0.68 (s)	3.8 (44.8%)	0.001	0.004
**Fatty acids**				
**All UFAs**	5.35 (m)	4.0 (44.0%)	0.001	0.004
**All PUFAs**	2.82 (m)	4.6 (42.3%)	0.001	0.004
**ω-6 PUFAs**	2.06 (m)	2.5 (55.1%)	0.003	0.008
**FFAs + FAs in 1-MGs**	2.35 (t)	−1.2 (90.9%)	0.045	0.048
**Glycerophospholipids**				
**Plasmalogens**	5.90 (d)	5.6 (40.3%)	0.001	0.004
**Glycerolipids**				
**1-MGs**	4.18 (ddd)	−1.3 (84%)	0.025	0.033
**TGs**	4.29 (dd)	11.9 (58.2%)	0.001	0.004
**Unassigned compounds**				
**U_0.83_**	0.83 (br)	−3.0 (78.3%)	0.001	0.004
**U_2.38_**	2.38 (m)	1.8 (66.8%)	0.013	0.023
**U_7.76_**	7.76 (s)	−2.3 (89.1%)	0.013	0.023

## Data Availability

Data to be made available in a publicly accessible repository that does not issue DOIs. Data will be found on the Metabolomics Workbench, https://www.metabolomicsworkbench.org/ (accessed on 2 March 2022) (submission under review), with data track id numbers 3173 (lipidomic data) and 3175 (exometabolome).

## Supplementary Materials

The following are available online at https://www.mdpi.com/article/10.3390/cells11081257/s1, Figure S1. Spectral comparison of the most evident differences between the initial and later days of osteoinduction; Figure S2. Typical ^1^H NMR spectra of conditioned media from hAMSC after 1 day of osteoinduction; Table S1. ^1^H NMR assignment of intracellular lipidic metabolites identified in hAMSCs during osteogenic differentiation; Table S2. Statistically significant lipidic changes throughout osteogenic differentiation of hAMSCs comparing consecutive timepoints analysed and extreme days; Table S3. ^1^H NMR assignment of metabolites identified in blank and hAMSCs conditioned osteogenic media samples throughout osteogenic differentiation.

## References

[1] Dominici M., Le Blanc K., Mueller I., Slaper-Cortenbach I., Marini F.C., Krause D.S., Deans R.J., Keating A., Prockop D.J., Horwitz E.M. (2006). Minimal criteria for defining multipotent mesenchymal stromal cells. The International Society for Cellular Therapy position statement. Cytotherapy.

[2] Iaquinta M.R., Mazzoni E., Bononi I., Rotondo J.C., Mazziotta C., Montesi M., Sprio S., Tampieri A., Tognon M., Martini F. (2019). Adult stem cells for bone regeneration and repair. Front. Cell Dev. Biol..

[3] Bispo D.S.C., Jesus C.S.H., Marques I.M.C., Romek K.M., Oliveira M.B., Mano J.F., Gil A.M. (2021). Metabolomic applications in stem cell research: A review. Stem Cell Rev. Rep..

[4] Coman C., Solari F.A., Hentschel A., Sickmann A., Zahedi R.P., Ahrends R. (2016). Simultaneous Metabolite, Protein, Lipid Extraction (SIMPLEX): A combinatorial multimolecular omics approach for systems biology. Mol. Cell. Proteom..

[5] Villaret-Cazadamont J., Poupin N., Tournadre A., Batut A., Gales L., Zalko D., Cabaton N.J., Bellvert F., Bertrand-Michel J. (2020). An optimized dual extraction method for the simultaneous and accurate analysis of polar metabolites and lipids carried out on single biological samples. Metabolites.

[6] Surrati A., Evseev S., Jourdan F., Kim D.-H., Sottile V. (2021). Osteogenic response of human mesenchymal stem cells analysed using combined intracellular and extracellular metabolomic monitoring. Cell. Physiol. Biochem..

[7] Casati S., Giannasi C., Niada S., Bergamaschi R.F., Orioli M., Brini A.T. (2021). Bioactive lipids in mscs biology: State of the art and role in inflammation. Int. J. Mol. Sci..

[8] Clémot M., Demarco R.S., Jones D.L. (2020). Lipid mediated regulation of adult stem cell behavior. Front. Cell Dev. Biol..

[9] Jurowski K., Kochan K., Walczak J., Barańska M., Piekoszewski W., Buszewski B. (2017). Analytical techniques in lipidomics: State of the art. Crit. Rev. Anal. Chem..

[10] Li J., Vosegaard T., Guo Z. (2017). Applications of nuclear magnetic resonance in lipid analyses: An emerging powerful tool for lipidomics studies. Prog. Lipid Res..

[11] Dai H., Hong B., Xu Z., Ma L., Chen Y., Xiao Y., Wu R. (2013). Nuclear magnetic resonance spectroscopy is highly sensitive for lipid-soluble metabolites. Neural Regen. Res..

[12] Bhinderwala F., Wase N., DiRusso C., Powers R. (2018). Combining mass spectrometry and NMR improves metabolite detection and annotation. J. Proteome Res..

[13] Kiamehr M., Viiri L.E., Vihervaara T., Koistinen K.M., Hilvo M., Ekroos K., Käkelä R., Aalto-Setälä K. (2017). Lipidomic profiling of patient-specific iPSC-derived hepatocyte-like cells. Dis. Model. Mech..

[14] Tanosaki S., Tohyama S., Fujita J., Someya S., Hishiki T., Matsuura T., Nakanishi H., Ohto-Nakanishi T., Akiyama T., Morita Y. (2020). Fatty acid synthesis is indispensable for survival of human pluripotent stem cells. iScience.

[15] Wu Y., Chen K., Xing G., Li L., Ma B., Hu Z., Duan L., Liu X. (2019). Phospholipid remodeling is critical for stem cell pluripotency by facilitating mesenchymal-to-epithelial transition. Sci. Adv..

[16] Lee H., Lee H.-R., Kim H.-Y., Lee H., Kim H.-J., Choi H.-K. (2019). Characterization and classification of rat neural stem cells and differentiated cells by comparative metabolic and lipidomic profiling. Anal. Bioanal. Chem..

[17] Rudan M.V., Mishra A., Klose C., Eggert U.S., Watt F.M. (2020). Human epidermal stem cell differentiation is modulated by specific lipid subspecies. Proc. Natl. Acad. Sci. USA.

[18] Li J., Cui Z., Zhao S., Sidman R.L. (2007). Unique glycerophospholipid signature in retinal stem cells correlates with enzymatic functions of diverse long-chain Acyl-CoA synthetases. Stem Cells.

[19] da Silva C.G., de Sá Barretto L.S., Lo Turco E.G., de Lima Santos A., Lessio C., Martins H.A., de Almeida F.G. (2020). Lipidomics of mesenchymal stem cell differentiation. Chem. Phys. Lipids.

[20] Rampler E., Egger D., Schoeny H., Rusz M., Pacheco M.P., Marino G., Kasper C., Naegele T., Koellensperger G. (2019). The power of LC-MS based multiomics: Exploring adipogenic differentiation of human mesenchymal stem/stromal cells. Molecules.

[21] Liaw L., Prudovsky I., Koza R.A., Anunciado-Koza R.V., Siviski M.E., Lindner V., Friesel R.E., Rosen C.J., Baker P.R.S., Simons B. (2016). Lipid profiling of *in vitro* cell models of adipogenic differentiation: Relationships with mouse adipose tissues. J. Cell. Biochem..

[22] Bojin F.M., Gruia A.T., Cristea M.I., Ordodi V.L., Paunescu V., Mic F.A. (2012). Adipocytes differentiated *in vitro* from rat mesenchymal stem cells lack essential free fatty acids compared to adult adipocytes. Stem Cells Dev..

[23] Gruia A.T., Suciu M., Barbu-Tudoran L., Azghadi S.M.R., Cristea M.I., Nica D.V., Vaduva A., Muntean D., Mic A.A., Mic F.A. (2016). Mesenchymal stromal cells differentiating to adipocytes accumulate autophagic vesicles instead of functional lipid droplets. J. Cell. Physiol..

[24] Alakpa E.V., Jayawarna V., Lampel A., Burgess K.V., West C.C., Bakker S.C.J., Roy S., Javid N., Fleming S., Lamprou D.A. (2016). Tunable supramolecular hydrogels for selection of lineage-guiding metabolites in stem cell cultures. Chem.

[25] Levental K.R., Surma M.A., Skinkle A.D., Lorent J.H., Zhou Y., Klose C., Chang J.T., Hancock J.F., Levental I. (2017). W-3 polyunsaturated fatty acids direct differentiation of the membrane phenotype in mesenchymal stem cells to potentiate osteogenesis. Sci. Adv..

[26] Orapiriyakul W., Tsimbouri M.P., Childs P., Campsie P., Wells J., Fernandez-Yague M.A., Burgess K., Tanner K.E., Tassieri M., Meek D. (2020). Nanovibrational stimulation of mesenchymal stem cells induces therapeutic reactive oxygen species and inflammation for three-dimensional bone tissue engineering. ACS Nano.

[27] Hodgkinson T., Monica Tsimbouri P., Llopis-Hernandez V., Campsie P., Scurr D., Childs P.G., Phillips D., Donnelly S., Wells J.A., O’Brien F.J. (2021). The use of nanovibration to discover specific and potent bioactive metabolites that stimulate osteogenic differentiation in mesenchymal stem cells. Sci. Adv..

[28] Georgi N., Cillero-Pastor B., Eijkel G.B., Periyasamy P.C., Kiss A., van Blitterswijk C., Post J.N., Heeren R.M.A., Karperien M. (2015). Differentiation of mesenchymal stem cells under hypoxia and normoxia: Lipid profiles revealed by time-of-flight secondary ion mass spectrometry and multivariate analysis. Anal. Chem..

[29] Rocha B., Cillero-Pastor B., Eijkel G., Bruinen A.L., Ruiz-Romero C., Heeren R.M.A., Blanco F.J. (2015). Characterization of lipidic markers of chondrogenic differentiation using mass spectrometry imaging. Proteomics.

[30] Kilpinen L., Tigistu-Sahle F., Oja S., Greco D., Parmar A., Saavalainen P., Nikkilä J., Korhonen M., Lehenkari P., Käkelä R. (2013). Aging bone marrow mesenchymal stromal cells have altered membrane glycerophospholipid composition and functionality. J. Lipid Res..

[31] Chatgilialoglu A., Rossi M., Alviano F., Poggi P., Zannini C., Marchionni C., Ricci F., Tazzari P.L., Taglioli V., Calder P.C. (2017). Restored *in vivo*-like membrane lipidomics positively influence *in vitro* features of cultured mesenchymal stromal/stem cells derived from human placenta. Stem Cell Res. Ther..

[32] Lu X., Chen Y., Wang H., Bai Y., Zhao J., Zhang X., Liang L., Chen Y., Ye C., Li Y. (2019). Integrated lipidomics and transcriptomics characterization upon aging-related changes of lipid species and pathways in human bone marrow mesenchymal stem cells. J. Proteome Res..

[33] Mastrangelo A., Panadero M.I., Perez L.M., Galvez B.G., Garcia A., Barbas C., Ruperez F.J. (2016). New insight on obesity and adipose-derived stem cells using comprehensive metabolomics. Biochem. J..

[34] Lee S.J., Yi T.G., Ahn S.H., Lim D.K., Kim S.-n., Lee H.J., Cho Y.K., Lim J.Y., Sung J.H., Yun J.H. (2018). Comparative study on metabolite level in tissue-specific human mesenchymal stem cells by an ultra-performance liquid chromatography quadrupole time of flight mass spectrometry. Anal. Chim. Acta.

[35] Burk J., Melzer M., Hagen A., Lips K.S., Trinkaus K., Nimptsch A., Leopold J. (2021). Phospholipid profiles for phenotypic characterization of adipose-derived multipotent mesenchymal stromal cells. Front. Cell Dev. Biol..

[36] DeVeaux S.A., Ogle M.E., Vyshnya S., Chiappa N.F., Leitmann B., Rudy R., Day A., Mortensen L.J., Kurtzberg J., Roy K. Characterizing human mesenchymal stromal cells’ immune-modulatory potency using targeted lipidomic profiling of sphingolipids.

[37] Lv M., Zhang S., Jiang B., Cao S., Dong Y., Cao L., Guo S. (2021). Adipose-derived stem cells regulate metabolic homeostasis and delay aging by promoting mitophagy. FASEB J..

[38] Surrati A., Linforth R., Fisk I.D., Sottile V., Kim D.H. (2016). Non-destructive characterisation of mesenchymal stem cell differentiation using LC-MS-based metabolite footprinting. Analyst.

[39] Amer M.H., Alvarez-Paino M., McLaren J., Pappalardo F., Trujillo S., Wong J.Q., Shrestha S., Abdelrazig S., Stevens L.A., Lee J.B. (2021). Designing topographically textured microparticles for induction and modulation of osteogenesis in mesenchymal stem cell engineering. Biomaterials.

[40] Sigmarsdottir T.B., McGarrity S., Yurkovich J.T., Rolfsson Ó., Sigurjónsson Ó.E. (2021). Analyzing metabolic states of adipogenic and osteogenic differentiation in human mesenchymal stem cells via genome scale metabolic model reconstruction. Front. Cell Dev. Biol..

[41] Bispo D.S.C., Jesus C.S.H., Correia M., Ferreira F., Bonifazio G., Goodfellow B.J., Oliveira M.B., Mano J.F., Gil A.M. NMR metabolomics assessment of osteogenic differentiation of adipose-tissue-derived mesenchymal stem cells. J. Proteome Res..

[42] Wu H., Southam A.D., Hines A., Viant M.R. (2008). High-throughput tissue extraction protocol for NMR- and MS-based metabolomics. Anal. Biochem..

[43] Kostidis S., Addie R.D., Morreau H., Mayboroda O.A., Giera M. (2017). Quantitative NMR analysis of intra- and extracellular metabolism of mammalian cells: A tutorial. Anal. Chim. Acta.

[44] Tukiainen T., Tynkkynen T., Mäkinen V.P., Jylänki P., Kangas A., Hokkanen J., Vehtari A., Gröhn O., Hallikainen M., Soininen H. (2008). A multi-metabolite analysis of serum by 1H NMR spectroscopy: Early systemic signs of Alzheimer’s disease. Biochem. Biophys. Res. Commun..

[45] Dais P., Misiak M., Hatzakis E. (2015). Analysis of marine dietary supplements using NMR spectroscopy. Anal. Methods.

[46] Subramanian A., Joshi B.S., Roy A.D., Roy R., Gupta V., Dang R.S. (2008). NMR spectroscopic identification of cholesterol esters, plasmalogen and phenolic glycolipids as fingerprint markers of human intracranial tuberculomas. NMR Biomed..

[47] Nieva-Echevarría B., Goicoechea E., Manzanos M.J., Guillén M.D. (2015). Usefulness of 1H NMR in assessing the extent of lipid digestion. Food Chem..

[48] Lefevre C., Panthu B., Naville D., Guibert S., Pinteur C., Elena-Herrmann B., Vidal H., Rautureau G.J.P., Mey A. (2019). Metabolic phenotyping of adipose-derived stem cells reveals a unique signature and intrinsic differences between fat pads. Stem Cells Int..

[49] Wishart D.S., Tzur D., Knox C., Eisner R., Guo A.C., Young N., Cheng D., Jewell K., Arndt D., Sawhney S. (2007). HMDB: The human metabolome database. Nucleic Acids Res..

[50] Veselkov K.A., Lindon J.C., Ebbels T.M.D., Crockford D., Volynkin V.V., Holmes E., Davies D.B., Nicholson J.K. (2009). Recursive segment-wise peak alignment of biological 1 H NMR spectra for improved metabolic biomarker recovery. Anal. Chem..

[51] Trygg J., Holmes E., Lundstedt T. (2007). Chemometrics in metabonomics. J. Proteome Res..

[52] Bridge P.D., Sawilowsky S.S. (1999). Increasing physicians’ awareness of the impact of statistics on research outcomes: Comparative power of the t-test and Wilcoxon rank-sum test in small samples applied research. J. Clin. Epidemiol..

[53] Berben L., Sereika S.M., Engberg S. (2012). Effect size estimation: Methods and examples. Int. J. Nurs. Stud..

[54] Benjamini Y., Hochberg Y. (1995). Controlling the false discovery rate: A practical and powerful approach to multiple testing. J. R. Stat. Soc. Ser. B.

[55] Mosconi E., Fontanella M., Sima D.M., Van Huffel S., Fiorini S., Sbarbati A., Marzola P. (2011). Investigation of adipose tissues in Zucker rats using *in vivo* and ex vivo magnetic resonance spectroscopy. J. Lipid Res..

[56] Rendina-Ruedy E., Guntur A.R., Rosen C.J. (2017). Intracellular lipid droplets support osteoblast function. Adipocyte.

[57] Gillet C., Spruyt D., Rigutto S., Dalla Valle A., Berlier J., Louis C., Debier C., Gaspard N., Malaisse W.J., Gangji V. (2015). Oleate abrogates palmitate-induced lipotoxicity and proinflammatory response in human bone marrow-derived mesenchymal stem cells and osteoblastic cells. Endocrinology.

[58] Else P.L. (2020). The highly unnatural fatty acid profile of cells in culture. Prog. Lipid Res..

[59] Casado-Díaz A., Santiago-Mora R., Dorado G., Quesada-Gómez J.M. (2013). The omega-6 arachidonic fatty acid, but not the omega-3 fatty acids, inhibits osteoblastogenesis and induces adipogenesis of human mesenchymal stem cells: Potential implication in osteoporosis. Osteoporos. Int..

[60] Abshirini M., Ilesanmi-Oyelere B.L., Kruger M.C. (2021). Potential modulatory mechanisms of action by long-chain polyunsaturated fatty acids on bone cell and chondrocyte metabolism. Prog. Lipid Res..

[61] Kashirina A.S., López-Duarte I., Kubánková M., Gulin A.A., Dudenkova V.V., Rodimova S.A., Torgomyan H.G., Zagaynova E.V., Meleshina A.V., Kuimova M.K. (2020). Monitoring membrane viscosity in differentiating stem cells using BODIPY-based molecular rotors and FLIM. Sci. Rep..

[62] Klontzas M.E., Vernardis S.I., Heliotis M., Tsiridis E., Mantalaris A. (2017). Metabolomics analysis of the osteogenic differentiation of umbilical cord blood mesenchymal stem cells reveals differential sensitivity to osteogenic agents. Stem Cells Dev..

[63] Klontzas M.E., Reakasame S., Silva R., Morais J.C.F., Vernardis S., MacFarlane R.J., Heliotis M., Tsiridis E., Panoskaltsis N., Boccaccini A.R. (2019). Oxidized alginate hydrogels with the GHK peptide enhance cord blood mesenchymal stem cell osteogenesis: A paradigm for metabolomics-based evaluation of biomaterial design. Acta Biomater..

[64] Li H., Guo H., Li H. (2013). Cholesterol loading affects osteoblastic differentiation in mouse mesenchymal stem cells. Steroids.

[65] Li K., Xiu C., Zhou Q., Ni L., Du J., Gong T., Li M., Saijilafu H.Y., Chen J. (2019). A dual role of cholesterol in osteogenic differentiation of bone marrow stromal cells. J. Cell. Physiol..

[66] Pike L.J., Han X., Chung K.-N., Gross R.W. (2002). Lipid rafts are enriched in arachidonic acid and plasmenylethanolamine and their composition is independent of caveolin-1 expression: A quantitative electrospray ionization/mass spectrometric analysis. Biochemistry.

[67] Baker N., Sohn J., Tuan R.S. (2015). Promotion of human mesenchymal stem cell osteogenesis by PI3-kinase/Akt signaling, and the influence of caveolin-1/cholesterol homeostasis. Stem Cell Res. Ther..

[68] van de Peppel J., van Leeuwen J.P.T.M. (2014). Vitamin D and gene networks in human osteoblasts. Front. Physiol..

[69] Zoeller R.A., Lake A.C., Nagan N., Gaposchkin D.P., Legner M.A., Lieberthal W. (1999). Plasmalogens as endogenous antioxidants: Somatic cell mutants reveal the importance of the vinyl ether. Biochem. J..

[70] Luthringer B.J.C., Katha U.M.R., Willumeit R. (2014). Phosphatidylethanolamine biomimetic coating increases mesenchymal stem cell osteoblastogenesis. J. Mater. Sci. Mater. Med..

[71] Butler W.T. (1989). The nature and significance of osteopontin. Connect. Tissue Res..

[72] Suzuki A., Iwata J. (2021). Amino acid metabolism and autophagy in skeletal development and homeostasis. Bone.

[73] Hu Y.Y., Rawal A., Schmidt-Rohr K. (2010). Strongly bound citrate stabilizes the apatite nanocrystals in bone. Proc. Natl. Acad. Sci. USA.

[74] Uno K., Takarada T., Takarada-Iemata M., Nakamura Y., Fujita H., Hinoi E., Yoneda Y. (2011). Negative regulation of osteoblastogenesis through downregulation of runt-related transcription factor-2 in osteoblastic MC3T3-E1 cells with stable overexpression of the cystine/glutamate antiporter xCT subunit. J. Cell. Physiol..

[75] Lin T.H., Yang R.S., Tang C.H., Wu M.Y., Fu W.M. (2008). Regulation of the maturation of osteoblasts and osteoclastogenesis by glutamate. Eur. J. Pharmacol..

[76] Takarada-Iemata M., Takarada T., Nakamura Y., Nakatani E., Hori O., Yoneda Y. (2011). Glutamate preferentially suppresses osteoblastogenesis than adipogenesis through the cystine/glutamate antiporter in mesenchymal stem cells. J. Cell. Physiol..

[77] Pattappa G., Heywood H.K., de Bruijn J.D., Lee D.A. (2011). The metabolism of human mesenchymal stem cells during proliferation and differentiation. J. Cell. Physiol..

[78] Chen C.-T., Shih Y.-R.V., Kuo T.K., Lee O.K., Wei Y.-H. (2008). Coordinated changes of mitochondrial biogenesis and antioxidant enzymes during osteogenic differentiation of human mesenchymal stem cells. Stem Cells.

[79] Shum L.C., White N.S., Mills B.N., De Mesy Bentley K.L., Eliseev R.A. (2016). Energy metabolism in mesenchymal stem cells during osteogenic differentiation. Stem Cells Dev..

[80] Zubiría M.G., Alzamendi A., Moreno G., Rey M.A., Spinedi E., Giovambattista A. (2016). Long-term fructose intake increases adipogenic potential: Evidence of direct effects of fructose on adipocyte precursor cells. Nutrients.

[81] Legeza B., Balázs Z., Odermatt A. (2014). Fructose promotes the differentiation of 3T3-L1 adipocytes and accelerates lipid metabolism. FEBS Lett..

[82] Felice J.I., Gangoiti M.V., Molinuevo M.S., McCarthy A.D., Cortizo A.M. (2014). Effects of a metabolic syndrome induced by a fructose-rich diet on bone metabolism in rats. Metabolism.

[83] Kim S.P., Li Z., Zoch M.L., Frey J.L., Bowman C.E., Kushwaha P., Ryan K.A., Goh B.C., Scafidi S., Pickett J.E. (2017). Fatty acid oxidation by the osteoblast is required for normal bone acquisition in a sex- and diet-dependent manner. JCI Insight.

[84] Bermeo S., Al Saedi A., Vidal C., Khalil M., Pang M., Troen B.R., Myers D., Duque G. (2019). Treatment with an inhibitor of fatty acid synthase attenuates bone loss in ovariectomized mice. Bone.

[85] Nováková S., Danchenko M., Okajčeková T., Baranovičová E., Kováč A., Grendár M., Beke G., Pálešová J., Strnádel J., Janíčková M. (2021). Comparative proteomic and metabolomic analysis of human osteoblasts, differentiated from dental pulp stem cells, hinted crucial signaling pathways promoting osteogenesis. Int. J. Mol. Sci..

[86] Newman J.C., Verdin E. (2014). Ketone bodies as signaling metabolites. Trends Endocrinol. Metab..

[87] Zhao Y., Zou B., Shi Z., Wu Q., Chen G.Q. (2007). The effect of 3-hydroxybutyrate on the *in vitro* differentiation of murine osteoblast MC3T3-E1 and *in vivo* bone formation in ovariectomized rats. Biomaterials.

[88] Crown S.B., Marze N., Antoniewicz M.R. (2015). Catabolism of branched chain amino acids contributes significantly to synthesis of odd-chain and even-chain fatty acids in 3T3-L1 adipocytes. PLoS ONE.

[89] Liu X., Cooper D.E., Cluntun A.A., Warmoes M.O., Zhao S., Reid M.A., Liu J., Lund P.J., Lopes M., Garcia B.A. (2018). Acetate production from glucose and coupling to mitochondrial metabolism in mammals. Cell.

[90] Meiser J., Tumanov S., Maddocks O., Labuschagne C.F., Athineos D., Van Den Broek N., Mackay G.M., Gottlieb E., Blyth K., Vousden K. (2016). Serine one-carbon catabolism with formate overflow. Sci. Adv..

[91] Pietzke M., Meiser J., Vazquez A. (2020). Formate metabolism in health and disease. Mol. Metab..

[92] Langenbach F., Handschel J. (2013). Effects of dexamethasone, ascorbic acid and β-glycerophosphate on the osteogenic differentiation of stem cells *in vitro*. Stem Cell Res. Ther..

[93] Schäck L.M., Noack S., Winkler R., Wißmann G., Behrens P., Wellmann M., Jagodzinski M., Krettek C., Hoffmann A. (2013). The phosphate source influences gene expression and quality of mineralization during *in vitro* osteogenic differentiation of human mesenchymal stem cells. PLoS ONE.

[94] Roberts S.J., Stewart A.J., Sadler P.J., Farquharson C. (2004). Human PHOSPHO1 exhibits high specific phosphoethanolamine and phosphocholine phosphatase activities. Biochem. J..

[95] Yadav M.C., Simão A.M.S., Narisawa S., Huesa C., McKee M.D., Farquharson C., Millán J.L. (2011). Loss of skeletal mineralization by the simultaneous ablation of PHOSPHO1 and alkaline phosphatase function: A unified model of the mechanisms of initiation of skeletal calcification. J. Bone Miner. Res..

