# Supplementary material for: Endo- and Exometabolome Crosstalk in Mesenchymal Stem Cells Undergoing Osteogenic Differentiation
Source: Cells. 2022 Apr 7;11(8):1257. doi: 10.3390/cells11081257 (PMC9024772; doi:10.3390/cells11081257)
Supplement: Supplementary file 1 [file cells-11-01257-s001.zip › cells-1644135-supplementary.pdf]

Figure S1

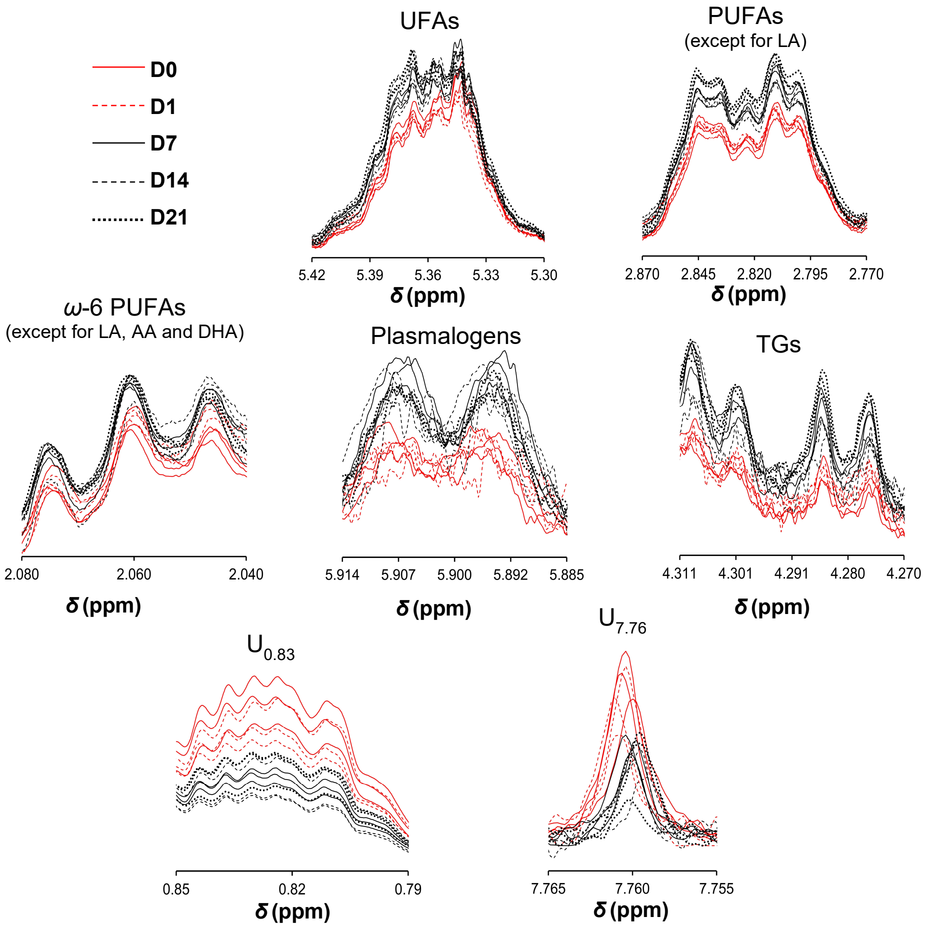

Figure S1. Spectral comparison of the most evident differences between the initial (days 0 and 1, in red) and later days of osteoinduction (days 7, 14 and 21, in black). AA, arachidonic acid; DHA, docosahexaenoic acid; LA, linoleic acid; PUFAs, polyunsaturated fatty acids; TGs, triacylglycerides; UFAs, unsaturated fatty acids. Di: day i; U_δ: unassigned signal at chemical shift δ.

Figure S2

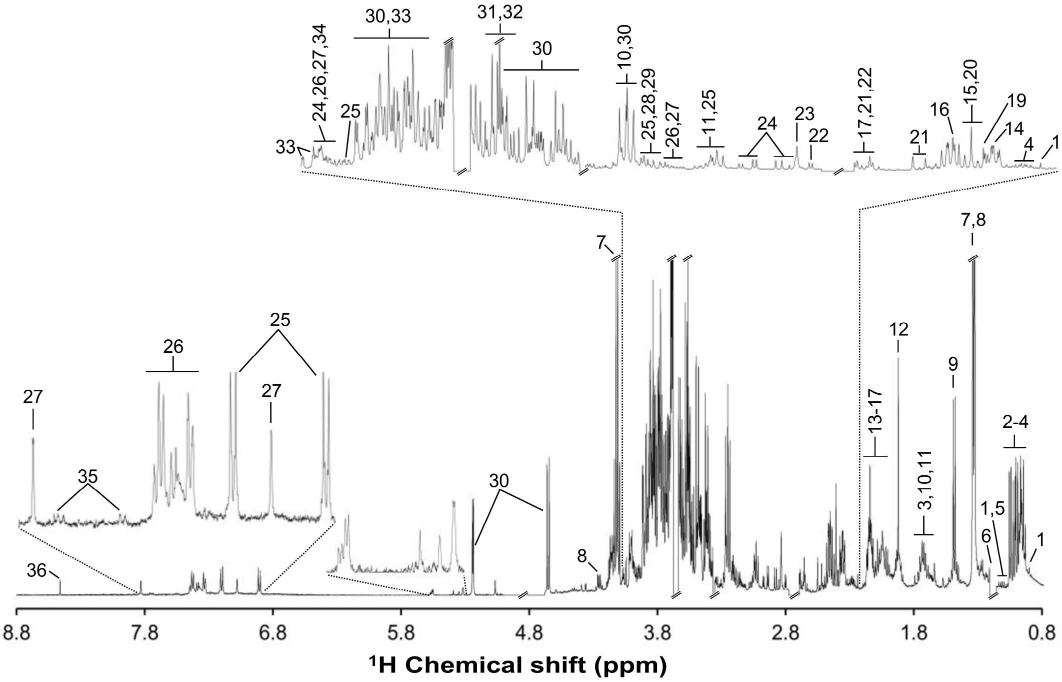

Figure S2. Typical 500 MHz ^{1}H NMR spectra of conditioned media from hAMSC after 1 day of exposure to osteogenic supplementation. Excluded spectral areas include the signals of water (δ 5.00 - 4.68), methanol (δ 3.37 - 3.35), ethanol (δ 3.67 - 3.64 and δ 1.21 - 1.16) (methanol and ethanol are contaminants resulting from the extraction procedure and material cleaning procedures, respectively) and dimethylamine (δ 7.77 – 2.70). Peak assignment: 1. 3-Methyl-2-oxovalerate, 2. Isoleucine, 3. leucine, 4. valine, 5. 3-hydroxyisobutyrate, 6. 3-hydroxybutyrate, 7. lactate, 8. threonine, 9. alanine, 10. arginine, 11. lysine, 12. acetate, 13. proline, 14. glutamate, 15. pyroglutamate, 16. glutamine, 17. methionine, 18. acetone, 19. pyruvate, 20. succinate, 21. citrate, 22. aspartate, 23. methylguanidine, 24. asparagine, 25. tyrosine, 26. phenylalanine, 27. histidine, 28. cystine, 29. choline, 30. glucose, 31. glycerol, 32. glycine, 33. fructose, 34. serine, 35. tryptophan, 36. formate. The complete list of assignments is present in Table S3.

Table S1. ^{1}H NMR assignment (500 MHz) of intracellular lipidic metabolites identified in hAMSCs during osteogenic differentiation, listed by increasing chemical shift value. †, tentative assignment; [a], Specific signals arising from DHA, EPA and AA could not be identified. Abbreviations: 1-MG, 1-monoacylgliceride; AA, arachidonic acid; DHA, docosahexaenoic acid; EPA, eicosapentaenoic acid; FAs, fatty acids; FFAs, free fatty acids; GPL, glycerophospholipids; LA, Linoleic acid; MUFAs, monounsaturated fatty acids; PtdCho, phosphatidylcholine; PtdEtn, phosphatidylethanolamine; PUFAs, polyunsaturated fatty acids; SM, sphingomyelin; TGs, triacylglyceride; UFAs, unsaturated fatty acids. Multiplicity: s, singlet; d, doublet; dd, doublet of doublets; ddd, doublet of doublets of doublets; t, triplet; q, quartet; m, multiplet; br, broad signal.

**Metabolite**		**δ ^{1}H in ppm (multiplicity, assignment)**
Cholesterol	Total	0.68 (s, 18-C$\underline{\mathbf{H}}_3$)
	Total	0.86 (d, 26-C$\underline{\mathbf{H}}_3$)
	Total	0.87 (d, 27-C$\underline{\mathbf{H}}_3$)
	Total	0.91 (d, 21-C$\underline{\mathbf{H}}_3$)
	Free	1.01 (s, 19-C$\underline{\mathbf{H}}_3$)
	Esterified	1.02 (s, 19-C$\underline{\mathbf{H}}_3$)
	Total	1.12 (m, multiple cholesterol protons)
	Total	1.48 (m, multiple cholesterol protons)
	Total	1.84 (m, multiple cholesterol protons)
	Total	1.96 (br, 7–C$\underline{\mathbf{H}}_2$/8–C$\underline{\mathbf{H}}$)
	Total	2.23 (br, 4-C$\underline{\mathbf{H}}_2$)
	Free	3.53 (br, 3-C$\underline{\mathbf{H}}$)
	Esterified	4.61 (m, 3-C$\underline{\mathbf{H}}$)
	Total	5.32 (br, 6-C$\underline{\mathbf{H}}$)
Fatty acids (FAs)	All FAs (except ω-3)	0.88 (t, C$\underline{\mathbf{H}}_3$)
	ω-3 FAs	0.97 (t, C$\underline{\mathbf{H}}_3$)
	All FAs	1.27 (m, (C$\underline{\mathbf{H}}_2$)$_n$)
	All FAs (except DHA, EPA and AA[a])	1.60 (m, -C$\underline{\mathbf{H}}_2$-CH$_2$-CO-)
	All UFAs	1.98-2.08 (m, -CH$_2$-C$\underline{\mathbf{H}}_2$-CH=)
	MUFAs	2.01 (m, -CH$_2$-C$\underline{\mathbf{H}}_2$-CH=)
	ω-3 and ω-6 FAs (except AA and DHA[a])	2.06 (m, -CH$_2$-C$\underline{\mathbf{H}}_2$-CH=)
	FAs in TG (except DHA[a])	2.29 (m, -C$\underline{\mathbf{H}}_2$-CO)
	FFAs + FAs in 1-MG (except DHA[a])	2.35 (t, -C$\underline{\mathbf{H}}_2$-CO)
	LA (18:2, ω-6)	2.77 (t, =CH-C$\underline{\mathbf{H}}_2$-CH=)
	PUFAs (except LA)	2.82 (m, =CH-C$\underline{\mathbf{H}}_2$-CH=)
	UFAs	5.35 (m, -$\underline{\mathbf{H}}$C=C$\underline{\mathbf{H}}$-)
Glycerophospholipids	PtdEtn	3.16 (br, N-C$\underline{\mathbf{H}}_2$ of ethanolamine)
(GPL) and	SMs	3.30 (s, -N$^+$(C$\underline{\mathbf{H}}_3$)$_3$)
sphingomyelins (SMs)	PtdCho	3.32 (s, -N$^+$(C$\underline{\mathbf{H}}_3$)$_3$ of choline)
	PtdCho + SMs	3.74 (br, N-C$\underline{\mathbf{H}}_2$ of choline)
	Plasmalogens †	3.85 (m, 1-C$\underline{\mathbf{H}}_2$ of glycerol)
	All GPL	3.95 (m, PO-(3-C$\underline{\mathbf{H}}_2$) of glycerol)
	PtdEtn	4.06 (br, PO-C$\underline{\mathbf{H}}_2$ of ethanolamine)
	All GPL	4.38 (m, 1-C$\underline{\mathbf{H}}_2$ of glycerol)
	Plasmalogens †	5.16 (m, 2-C$\underline{\mathbf{H}}_2$ of glycerol)
	All GPL	5.22 (m, 2-C$\underline{\mathbf{H}}$ of glycerol)
	SMs	5.68 (m, -CH$_2$-C$\underline{\mathbf{H}}$=CH-CHOH-)
	Plasmalogens	5.90 (d, O-C$\underline{\mathbf{H}}$=CH)
Glycerolipids	1-MGs	3.65 (ddd, 3-C$\underline{\mathbf{H}}_2$ of glycerol)
	TGs	4.15 (dd, 1-C$\underline{\mathbf{H}}_2$/3-C$\underline{\mathbf{H}}_2$ of glycerol)
	1-MGs	4.18 (ddd, 1-C$\underline{\mathbf{H}}_2$ of glycerol)
	TGs	4.29 (dd, 1-C$\underline{\mathbf{H}}_2$/3-C$\underline{\mathbf{H}}_2$ of glycerol)
	TGs	5.28 (m, 2-C$\underline{\mathbf{H}}$ of glycerol)

Table S2. Statistically significant lipidic changes throughout osteogenic differentiation of hAMSCs comparing consecutive timepoints analyzed (from day 0, and until day 21) and extreme days (day 0 *vs.* 21). Effect size (ES) values and corresponding errors were calculated according to reference [53] (level increases and decreases in the later days are represented by positive and negative ES values). As in Table 1, all differences presented were confirmed by visual inspection of the spectra and are statistically significant (Wilcoxon Rank-sum test p-values < 0.05) [52]. However, these *p*-values have lower statistical bearing due to the low number of samples per day. Benjamini-Hochberg false discovery rate (FDR) [54] correction was applied for multiple testing, and none of the FDR adjusted *p*-values remained statistically significant. [a], peak used for integration (part of the spin system). D_i: day i; U_δ: unassigned signal at chemical shift δ. Lipid and multiplicity abbreviations as defined in Table S1.

**Metabolite**	**δ ¹H (multiplicity)** [a]	**Effect size (ES error %)**				
		D_0 *vs.* D_1	D_1 *vs.* D_7	D_7 *vs.* D_{14}	D_{14} *vs.* D_{21}	D_0 *vs.* D_{21}
**Cholesterol**						
Total	0.68 (s)	-	4.0 (69.4)	-	-	-
Free	1.01 (s)	-	-	-	-2.1 (94.4)	-4.9 (65.3)
**Fatty acids**						
All UFAs	5.35 (m)	-	7.3 (60.7)	-	-	5.4 (63.9)
All PUFAs	2.82 (m)	-	9.8 (58.9)	-	-	10 (58.8)
ω-6 PUFAs	2.06 (m)	-	1.9 (100.7)	-	-	4.9 (65.4)
MUFAs	2.01 (m)	-2.7 (81.7)	4.2 (68.4)	-	-	-
FAs in TGs + GPL	2.29 (m)	-2.5 (86.4)	4.7 (66.1)	-	-	-
FFAs + FAs in 1-MGs	2.35 (t)	1.8 (103.7)	-3.5 (72.8)	-	-	-
**Glycerophospholipids & sphingolipids**						
PtdCho	3.32 (s)	-2.8 (80.4)	2.9 (78.7)	-	-	-
PtdEtn	3.16 (br)	-	3.6 (71.9)	2.3 (88.8)	-	-
Plasmalogens	5.90 (d)	-	7.7 (60.3)	-	-	9.1 (59.3)
SM	3.30 (s)	-	3.7 (71.5)	-	-	-
**Glycerolipids**						
1-MG	4.18 (ddd)	2.2 (92.2)	-4.5 (66.9)	-	-	-
TGs	4.29 (dd)	-	5.8 (63.0)	-	4.8 (65.5)	11.9 (58.2)
**Unassigned compounds**						
$U_{0.83}$	0.83 (br)	-	-3.8 (70.6)	-	-	-3.0 (78.3)
$U_{2.38}$	2.38 (br)	-	-	-	-	5.8 (63.0)
$U_{7.76}$	7.76 (s)	-	-	-	-	-2.3 (89.1)

Table S3. ^{1}H NMR assignment of metabolites identified in fresh (blank) and hAMSCs conditioned osteogenic media samples throughout osteogenic differentiation. Multiplicity: s, singlet; d, doublet; dd, doublet of doublets; dt, doublet of triplets; t, triplet; q, quartet; m, multiplet. The second column indicates the metabolite ID numbers found in both the Human Metabolome Database (HMDB) [49] and the Kyoto encyclopedia of genes and genomes (KEGG, https://www.genome.jp/kegg/). [a] tentative assignment; [b] metabolites included in the composition of minimum essential alpha medium (α-MEM); [c] signals arising from the osteogenic supplements.

**Metabolite**	**HMDB ID, KEGG ID**	**δ ^{1}H in ppm (multiplicity, assignment)**
3-Hydroxybutyrate (3-HIB)	HMDB0000011, C01089	1.20 (d, γ-C$\underline{H}_3$), 2.40 (dd, ½ α-C$\underline{H}_2$), 4.12 (m, β-C$\underline{H}$)
3-Hydroxyisobutyrate (3-HIBA) [a]	HMDB0000023, C06001	1.07 (d, C$\underline{H}_3$)
3-Methyl-2-oxovalerate (3M2OV) [a]	HMDB0000491, C03465	0.90 (t, C$\underline{H}_3$(CH$_2$)$_2$CO), 1.10 (d, C$\underline{H}_3$CH$_2$CO)
Acetate	HMDB0000042, C00033	1.92 (s, β-C$\underline{H}_3$)
Acetone	HMDB0001659, C00207	2.24 (s, α- C$\underline{H}_3$)
Alanine [b]	HMDB0000161, C00041	1.48 (d, β-C$\underline{H}_3$), 3.79 (q, α-C$\underline{H}$)
Arginine [b]	HMDB0000517, C00062	1.67 (m, ½ γ-C$\underline{H}_2$), 1.92 (m, β-C$\underline{H}_2$), 3.25 (t, δ-C$\underline{H}_2$), 3.76 (t, α-C$\underline{H}$)
Ascorbate [b,c]	HMDB0000044, C00072	4.52 (d, C**H** lactone ring)
Asparagine [b]	HMDB0000168, C00152	2.86/2.96 (dd/dd, β-C$\underline{H}_2$), 4.00 (dd, α-C$\underline{H}$)
Aspartate [b]	HMDB0000191, C00049	2.68/2.82 (dd/dd, β-C$\underline{H}_2$), 3.90 (dd, α-C$\underline{H}$)
Citrate	HMDB0000094, C00158	2.53/2.67 (d/d, C$\underline{H}_2$)
Choline [b]	HMDB0000097, C00114	3.21 (s, N(C$\underline{H}_3$)$_3$)
Creatine [a]	HMDB0000064, C00300	3.04 (s, N-C$\underline{H}_3$)
Cystine [b]	HMDB0000192, C00491	3.19/3.39 (dd/dd, β-C$\underline{H}_2$-S), 4.10 (dd, α-C$\underline{H}$)
Dimethylamine (DMA) [c]	HMDB0000087, C00543	2.73 (s, C$\underline{H}_3$)
Formate	HMDB0000142, C00058	8.46 (s, C$\underline{H}$)
Fructose	HMDB0000660, C00095	4.00 (m, 5-C$\underline{H}$), 4.03 (m, 6-C$\underline{H}_2$)
α-Glucose [b]	HMDB0003345, C00031	3.42 (t, 4-C$\underline{H}$), 3.54 (dd, 2-C$\underline{H}$), 3.72 (t, 3-C$\underline{H}$), 3.86 (m, ½ 6-C$\underline{H}_2$), 3.84 (m, 5-C$\underline{H}$), 5.24 (d, 1-C$\underline{H}$)
β-Glucose [b]	HMDB0000122, C00221	3.25 (m, 2-C$\underline{H}$), 3.41 (t, 4-C$\underline{H}$), 3.47 (m, 5-C$\underline{H}$), 3.50 (t, 3-C$\underline{H}$), 3.73/3.90 (dd, 6-C$\underline{H}_2$), 4.65 (d, 1-C$\underline{H}$)
Glutamate [b]	HMDB0000148, C00025	2.06/2.13 (m/m, β-C$\underline{H}_2$), 2.35 (m, γ-C$\underline{H}_2$), 3.75 (dd, α-C$\underline{H}$)
Glutamine [b]	HMDB0000641, C00064	2.14 (m, β-C$\underline{H}_2$), 2.46 (m, γ-C$\underline{H}_2$), 3.77 (t, α-C$\underline{H}$)
Glycerol	HMDB0000131, C00116	3.56/3.66 (dd/dd, 1-C$\underline{H}_2$ & 3-C$\underline{H}_2$), 3.78 (m, 2-C$\underline{H}$)
β-Glycerophosphate [c]	HMDB0002520, C02979	3.69 (m, 1-C$\underline{H}_2$/3-C$\underline{H}_2$), 4.16 (m, 2-C$\underline{H}$)
Glycine [b]	HMDB0000123, C00037	3.56 (s, α-C$\underline{H}_2$)
Histidine [b]	HMDB0000177, C00135	3.14/3.25 (dd/dd, β -C$\underline{H}_2$), 4.00 (dd, α-C$\underline{H}_2$), 7.08 (s, 5-C$\underline{H}$ ring), 7.84 (s, 2-C$\underline{H}$ ring)
Isoleucine [b]	HMDB0000172, C00407	0.94 (t, δ-C$\underline{H}_3$), 1.02 (d, γ′-C$\underline{H}_3$), 1.27/1.47 (m/m, γ-C$\underline{H}_2$), 1.99 (m, β-C$\underline{H}$), 3.67 (d, α-C$\underline{H}$)
Lactate	HMDB0000190, C00186	1.33 (d, C$\underline{H}_3$), 4.11 (q, C$\underline{H}$)
Leucine [b]	HMDB0000687, C00123	0.96/0.97 (d/d, δ-C$\underline{H}_3$), 1.72 (m, γ-C$\underline{H}$ & β-C$\underline{H}_2$), 3.74 (m, α-C$\underline{H}$)
Lysine [b]	HMDB0000182, C00047	1.48 (m, γ-C$\underline{H}_2$), 1.72 (m, δ-C$\underline{H}_2$), 1.92 (m, β-C$\underline{H}_2$), 3.03 (t, ε-C$\underline{H}_2$, t), 3.73 (t, α-C$\underline{H}$)
Methionine [b]	HMDB0000696, C00073	2.14/2.20 (m/m, β-$\underline{H}_2$), 2.14 (s, C$\underline{H}_3$), 2.65 (t, γ-C$\underline{H}_2$)
Methylguanidine	HMDB0001522, C02294	2.83 (s, C$\underline{H}_3$)
myo-inositol [b]	HMDB0000211, C00137	3.28 (t, 5-C$\underline{H}$), 4.07 (t, 2-C$\underline{H}$)
Phenylalanine [b]	HMDB0000159, C00079	3.14/3.29 (dd/dd, β-C$\underline{H}_2$), 7.33 (m, 2-C$\underline{H}$ & 6-C$\underline{H}$ ring), 7.38 (m, 4-C$\underline{H}$ ring), 7.43 (m, 3-C$\underline{H}$ & 5-C$\underline{H}$ ring)
Proline [b]	HMDB0000162, C00148	2.03 (m, γ-C$\underline{H}_2$), 2.04/2.35(m/m, β-C$\underline{H}_2$),

		3.35/3.42 (dt/dt, δ-C**H**$_2$), 4.15 (dd, α-C**H**)
Pyroglutamate	HMDB0000267, C01879	2.41(m, γ-C**H**$_2$), 2.51 (m, ½ β-C**H**$_2$), 4.19 (dd, α-C**H**)
Pyruvate [b]	HMDB0000243, C00022	2.38 (s, C**H**$_3$)
Serine [a,b]	HMDB0000187, C00065	3.95/3.99 (dd/dd, β-C**H**$_2$)
Succinate	HMDB0000254, C00042	2.41 (s, C**H**$_2$)
Threonine [b]	HMDB0000167, C00188	1.32 (d, γ-C**H**$_3$), 3.58 (d, β-C**H**), 4.26 (dd, α-C**H**)
Tryptophan [b]	HMDB0000929, C00078	7.29 (t, η^2-C**H** indole ring), 7.55 (d, ξ^2-C**H** indole ring), 7.74 (d, ε^3-C**H** indole ring)
Tyrosine [b]	HMDB0000158, C00082	6.91 (d, 3-C**H** & 5-**H** ring), 7.20 (d, 2-C**H** & 6-**H** ring)
Valine [b]	HMDB0000883, C00183	1.00 (d, γ-C**H**$_3$), 1.05 (d, γ′-C**H**$_3$), 2.28 (m, β-C**H**), 3.61 (d, α-C**H**)
